# Interferon-gamma-inducible protein 30 prevents IFN-γ-receptor 1 degradation to maintain PD-L1 and MHC-II levels in metastatic melanoma

**DOI:** 10.1186/s12964-026-02710-9

**Published:** 2026-02-12

**Authors:** Shodai Mizuno, Yuka Mizuno, Kodai Abe, Anne M. Macy, Kelly K. Chong, Yuta Kobayashi, Karen T. Hastings, Dave S. B. Hoon, Matias A. Bustos

**Affiliations:** 1 Department of Translational Molecular Medicine, Saint John’s Cancer Institute (SJCI), Providence Saint John’s Health Center (SJHC), Santa Monica, CA 90404 USA; 2 Phoenix Veterans Affairs Health Care System, Phoenix, AZ USA; 3 Department of Dermatology, College of Medicine - Phoenix, University of Arizona, Phoenix, AZ USA; 4 University of Arizona Cancer Center, University of Arizona, Tucson, AZ USA; 5 Department of Genome Sequencing Center, SJCI, Providence SJHC, Santa Monica, CA 90404 USA

**Keywords:** IFI30, CTSL, HLA-DR, CTSS, PD-L1, CMTM6, HIP1R, MHC-II, Immune checkpoint inhibitors

## Abstract

**Background:**

Immunotherapies such as immune checkpoint inhibitors (ICIs) targeting programmed death protein 1 (PD-1) and programmed death-ligand 1 (PD-L1) have successfully improved outcomes in metastatic melanoma (MM) patients. PD-L1 and major histocompatibility complex class II (MHC-II) levels in tumor cells are critical in modulating ICI responses; however, the regulatory mechanisms controlling PD-L1 and MHC-II expression levels are still not fully characterized.

**Methods:**

Targeted mRNA sequencing data comparing tissue samples from MM patients (*n* = 25). Publicly available RNA-Seq [TCGA-SKCM (*n* = 383), PMID31792460 (*n* = 121), PRJEB23709 (*n* = 73)] and proteomic [PXD006003 (*n* = 63)] datasets from MM patients were utilized for bioinformatic analysis. Functional assays were performed on MM cell lines and multiplex immunofluorescence on tumor samples from MM patients to validate in-silico observations.

**Results:**

Here, we showed that interferon gamma (IFN-γ) inducible factor 30 (IFI30) has a dual role in preventing PD-L1 and MHC-II lysosomal degradation mediated by cathepsin L (CTSL), and modulating IFN-γ pathway signaling. Briefly, IFI30, PD-L1, MHC-II and CTSL levels are stimulated by IFN-γ in MM cell lines. The basal and IFN-γ-stimulated protein/mRNA levels of PD-L1 and MHC-II dramatically decreased, while CTSL levels increased in MM with *IFI30* knockdown. Blockage of lysosome acidification prevented PD-L1 protein degradation in MM with *IFI30* knockdown. Conversely, *CTSL* knockdown significantly increased IFI30, PD-L1 and MHC-II levels. *IFI30* knockdown decreased the levels of IFN-γ receptor 1 (IFNGR1) at the plasma membrane, blocked IFN-γ pathway downstream signaling, and decreased PD-L1 and MHC-II mRNA/protein levels. *IFNGR1* knockdown in MM cells resembled the phenotype observed for *IFI30* knockdown. Of clinical relevance, MM patients with high-*IFI30* levels in tumor tissue samples showed a better progression-free survival and better responses to ICIs in three independent datasets (PMID31792460, PRJEB23709, and PXD006003). High-*IFI30* levels in tumor tissue samples were associated with increased infiltration levels of M1 macrophages, CD8^+^ and CD4^+^ T cells.

**Conclusions:**

IFI30 exerts a negative modulation on CTSL to regulate IFNGR1, PD-L1, and MHC-II levels during IFN-γ stimulation. IFI30 levels may represent a key regulatory factor of IFN-γ pathway associated with ICI responses in MM patients.

**Supplementary Information:**

The online version contains supplementary material available at 10.1186/s12964-026-02710-9.

## Background

Immune checkpoint inhibitors (ICIs) targeting programmed cell death 1 (PD-1) or programmed death-ligand 1 (PD-L1) inhibitors represent the standard of care and are the most effective immunotherapies for metastatic melanoma (MM) patients [1]. However, ICI treatment does not always provide sustained clinical benefit and MM patients treated with PD-1 inhibitors have a short median progression-free survival (PFS) ranging from 6 to 8 months [2, 3]. Also, ~ 20% of MM patients carry the risk of severe grade 3–4 immune-related adverse events [1]. PD-L1 levels on tumor cells measured by immunohistochemistry (IHC) were previously reported to be a potential biomarker for response to PD-1/PD-L1 inhibitors [4]. However, clinical trials showed that high PD-L1 levels do not always correlate with the efficacy of ICIs targeting PD-1/PD-L1 and indeed, patients with low or negative PD-L1 levels may still benefit from treatment with ICIs [4, 5]. There is an unmet need to establish reliable biomarkers that can predict clinical efficacy of PD-1/PD-L1 inhibitors in MM to improve clinical outcomes.

The rationale for the usage of ICI relies on the immunosuppressive regulatory effects as a consequence of the interaction between PD-L1 (on tumor cells) and PD-1 (on T cells) [6]. Recent reports have revealed several mechanisms that control the expression levels of PD-L1 in tumor cells: (1) transcriptional regulation of PD-L1; (2) post-translational modifications on PD-L1; and (3) molecular mechanisms controlling PD-L1 transportation to the plasma membrane and from the plasma membrane to recycling endosomes and lysosomes for proteolytic degradation [7–9]. However, the regulatory events controlling PD-L1 levels in MM required further characterization, particularly in the context of interferon gamma (IFN-γ) stimulation.

IFN-γ inducible factor 30 (IFI30) – most commonly known as Interferon gamma (IFN-γ) inducible lysosomal thiol reductase (IFI30) – is localized in endosomes, lysosomes, and phagosomes [10]. IFI30 is required for efficient major histocompatibility complex class II (MHC-II) restricted presentation of multiple melanoma antigens[11, 12]; however, this function is generally limited to professional antigen presenting cells (APCs), including B cells, monocytes/macrophages, and bone marrow-derived dendritic cells [11, 12]. IFI30 is induced by IFN-γ and detected in melanoma cells (58–70%) of MM tissue samples [13]. Previous studies showed that bulk high-*IFI30*mRNA levels are associated with improved overall survival (OS) in MM patients [14], and high IFI30 protein levels in melanoma cells is associated with improved OS in ICI-treated MM patients [13]. Additionally, melanoma cell-specific expression of MHC-II is associated with improved response to PD-1/PD-L1 inhibitors [15, 16]. Therefore, the purpose of this study is to elucidate the molecular mechanisms controlled by IFI30 during IFN-γ stimulation that influences PD-L1 and MHC-II levels, which could explain the associations with clinical outcomes and treatment responses.

Consistent with previous observations in MM patients, this study found that *IFI30* mRNA levels were significantly elevated in lymph node metastasis (LNM) tissues from MM patients using next-generation sequencing (NGS)-based targeted transcriptomic analysis. Functionally, *IFI30* depletion showed to have a dual regulation: (1) to promote the cathepsin-L (CTSL)-mediated protein degradation of PD-L1 and Human leukocyte antigen - DR isotype (HLA-DR) protein levels, upon IFN-γ stimulation, and (2) to control PD-L1 and HLA-DR transcriptional regulation by targeting IFNGR1 for degradation, which suppresses IFN-γ signaling pathway activation. Of clinical relevance, low-IFI30 protein/mRNA levels were correlated with low levels of PD-L1 and HLA-DR, as well as a decreased immune infiltration (CD4^+^, CD8^+^ T cells, and M1 macrophages) in pre-treatment tissue samples of MM patients receiving ICIs. In silico and multiplex immunofluorescence analyses demonstrated that tumor samples of MM patients who responded to PD-L1 inhibitors had significantly higher levels of IFI30, suggesting potential clinical implications for MM patients under ICI treatment.

## Materials and methods

### Patient selection

The study was conducted following the Declaration of Helsinki. This study involves human participants and was approved by Ethics Committees: SJHC/SJCI Joint Institutional Review Board (IRB). Approval ID#: MORD-RTPCR-0995. The study included two cohorts of MM patients. The first cohort consisted of MM patients who underwent surgery. formalin-fixed-paraffin-embedded (FFPE) tissues samples utilized were pathologically diagnosed as LNM positive (LNM^+^, *n* = 17) or LNM negative (LNM^−^, *n* = 8) at Providence Saint John’s Health Center (PSJHC). Only tissues obtained from LNM were included in the analysis. Patients who had received any preoperative therapy were excluded. The clinical and pathological information collected from each is described in Table S1. The second cohort consisted of FFPE LNM from patients who underwent surgery and were pathologically diagnosed with stage III/IV metastatic melanoma (*n* = 12) at PSJHC. Patients who had received any preoperative therapy were excluded from the analysis. Only tissues obtained from LNM were included in the analysis. The clinical and pathological information collected from each is described in Table S2. All FFPE sections were evaluated using hematoxylin and eosin (H&E) staining by a board-certified pathologist expert in melanoma diagnosis at the Surgery Pathology Department. at SJHC.

### HTG EdgeSeq autoimmune panel

**Fig. 1:**
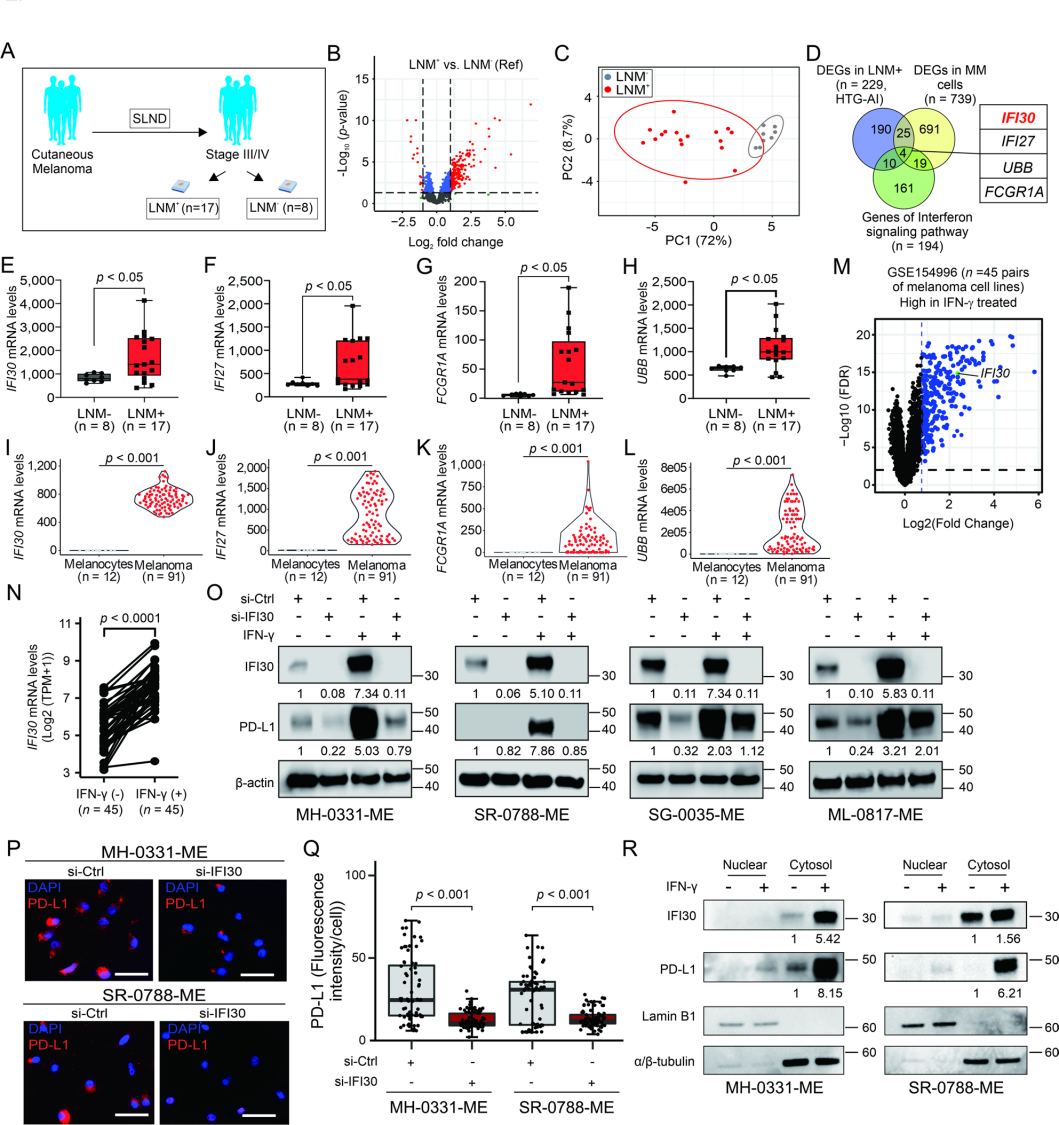
The *IFI30* mRNA levels are increased in melanoma LNM. **A** The schema of the cutaneous melanoma lymph node metastases (LNM) analyzed by HTG-AI panel: LNM^+^ compared to LNM^−^after sentinel-lymph node dissection (SLND). **B** Volcano plot shows the differentially expressed genes (DEGs) in melanoma LNM^+^ compared to LNM^−^. Four genes overlapped across the comparisons: *IFI30*, *IFI27*, *UBB*, *FCGR1A*. **C** Principal component analysis (PCA) plot comparing LNM^+^ and LNM^−^ tissue samples. **D** Venn diagram shows the overlapping DEGs among the upregulated genes in LNM^+^ from the HTG-AI dataset, the upregulated genes in metastatic melanoma (MM) cells obtained from public PRJEB23709 dataset, and interferon signaling pathway related genes. **E**-**H** Bar graphs show *IFI30* (**E**), *IFI27* (**F**) *FCGR1A* (**G**), and *UBB* (**H**) mRNA levels are in LNM^+^. **I**-**L** Violin plots show *IFI30* (**I**), *IFI27* (**J**) *FCGR1A* (**K**), and *UBB* (**L**) mRNA levels in MM cells from PRJEB23709 dataset after CODEFACs deconvolution analyses compared to normal melanocytes from GSE227015 datasets. **M** Volcano plot comparing the mRNA levels in 45 melanoma parental cell lines untreated (IFN-γ (-)) or treated with (IFN-γ (+)) for 6 h. **N** Comparison of *IFI30* mRNA levels between untreated or IFN-γ treated melanoma cell lines. **O** Western blot images show the levels of IFI30 and PD-L1 levels in MM cell lines control (si-Ctrl) or *IFI30* knockdown (si-*IFI30*) untreated or treated with IFN-γ. **P** Representative immunofluorescence images of PD-L1 in MM cell lines control (si-Ctrl) or with *IFI30* knockdown (si-IFI30) that were treated with IFN-γ. Scale bar = 50 μm. **Q** Quantification of PD-L1 (fluorescence intensity/cell) images shown in **P**. **R** Western blot images show the levels of IFI30 and PD-L1 in the cytosol and nuclear fractions of melanoma cells after IFN-γ stimulation. α/β-tubulin and lamin B1 were used as control of nuclear and cytosolic subcellular fractions, respectively. Data represents the mean ± SD. Significance was determined by T-test in (**E-L, Q**) and by Wilcoxon U-test in (**N**)

The analysis was performed in 5 μm sections from FFPE tissue samples (LNM^+^ (*n* = 17) and LNM^−^ (*n* = 8)) were obtained from MM patients who received surgery at SJHC (Fig. 1A). Detailed information for sample processing, library preparation, quality control, normalization, pooling, and profiling is described in Supplementary information_01. Briefly, for each tissue, a single section was processed and analyzed to measure the mRNA levels of 2,002 genes using the HTG EdgeSeq AutoImmune Panel (HTG-AI) assay [17, 18]. The HTG-AI transcriptomic data were compared between LNM^+^ and LNM^−^ groups using DESeq2 (1.38.3) and Log_2_ fold change (FC) |2| FDR < 0.01.

### Cell lines

LNM-derived cell lines from MM patients established at SJCI/JWCI (MH-0331-ME, SR-0788-ME, SG-0035-ME, VN-0326-ME, M15, M204, MB-1133-ME, ML-0817-ME, BO-0362-ME, CB-1147-ME, M211, SE-0154, AJL-0704, and HM-0525) were cultured in RPMI-1640 and supplemented with 10 mM HEPES, 10% heat-inactivated fetal bovine serum (FBS) and 1% penicillin-streptomycin. All experiments were performed with mycoplasma-free cell lines.

### Western blot

Traditional western blot was performed as previously described [19–21], except for the antibodies utilized that are summarized in Table S2. Western blot bands were visualized on an iBright FL 1500 Imager (Thermo Fisher Scientific, CA, USA), and all images were analyzed with ImageJ software (http://imagej.nih.gov/ij/). All the uncropped western blot images were included in Supplementary information_03.

### Biostatistical analysis

All statistical analyses were performed in R (version 4.2.1) using two-tailed tests. The distribution and variation within each group of data were assessed before selecting the correct statistical analysis. Fisher’s exact test or *Chi*-square test were applied when comparing nominal variables. Student’s t-test, linear mixed model, or Mann-Whitney U test were used for comparison between two groups. For comparisons between paired samples, the Wilcoxon signed-rank test was used. Correlations were determined by Spearman’s correlation test. All the figures were generated using the R package ggplot2 (version 3.4.4) and unified using Adobe Illustrator (Adobe Inc., Los Angeles, CA) or CorelDraw (Alludo, Ottawa, ON). All data were presented as mean ± standard error mean (SEM) or median (range).

Additional data for M&M can be found in the Supplementary_information_01–03 files.

## Results

### IFI30 is significantly upregulated in MM

To determine differentially expressed genes (DEGs) in LNM, we analyzed 25 lymph node (LN) tissue samples obtained from MM patients who underwent sentinel lymph node dissection at SJHC [22, 23]. The 25 LN tissue samples were stratified based on the presence of MM into LNM^+^ and LNM^−^ groups, after pathological confirmation of LN status (Fig. 1A). One single section from each of the 25 LN was profiled using HTG-AI panel to measure the mRNA levels of 2,002 genes (Fig. 1A-B). A total of 229 DEGs were found in LNM^+^ tissue samples (Fig. 1B, and Table S6), and the 229-mRNA signature distinguished LNM^+^ from LNM^−^ in a principal component analysis (PCA, Fig. 1C). To validate the results, we utilized the RNA-Seq data from PRJEB23709 dataset for 91 pre-treatment tissue samples obtained from MM patients treated with ICIs (anti-PD-1 ± anti-CTLA-4)[24]. The RNA-Seq data was deconvoluted using CODEFACS analysis as described in M&M. Then, the mRNA profiles of MM cells (PRJEB23709) were compared to the mRNA profiles obtained from melanocyte cells (GSE227015, Fig. S1A-C). An mRNA signature of 739 DEGs was found in MM cells (Fig. S1C, and Table S7). Given the importance of the IFN-γ pathway genes (Table S8) in MM patients and the relation to ICI treatment, we determined which DEGs related to the IFN-γ pathway were shared in the two separate comparisons —MM cells versus melanocytes and LNM⁺ versus LNM⁻— (Fig. 1D, Table S9-11). Of note *IFI30*, *IFI27*, *UBB*, and *FCGR1A* are IFN-γ pathway-related genes that were found commonly upregulated in tissue samples of MM patients as well as in deconvoluted melanoma cells of MM patients (Fig. 1D-L). The levels of *IFI30* were further assessed in the TCGA-SKCM in primary melanoma (PRM) and MM tumor samples. The results showed a significant upregulation in *IFI30*mRNA levels (Fig. S1D). In summary, melanoma LNM had gene signatures that were significantly upregulated and associated with IFN-γ signaling pathway. Based on these findings and previous literature supporting a role for IFI30 in MM[11–14, 25–34], we decided to investigate the function of IFI30 in the context of IFN-γ signaling pathway activation using MM cell lines derived from LNM.

### IFI30 and PD-L1 consistently increase after IFN-γ stimulation

IFI30 is a lysosomal thiol reductase commonly found in endosomes, lysosomes, and phagosomes[10]; however, the molecular functions of IFI30 in the context of IFN-γ signaling pathway activation are still not fully characterized. Using RNA-Seq data (GSE154996) obtained from melanoma cell lines treated with IFN-γ and control conditions, we observed that the *IFI30* mRNA levels were significantly increased in melanoma cell lines treated with IFN-γ (Fig. 1M-N). In a cohort of fourteen LNM-derived cell lines, the protein levels of IFI30 were variable under basal conditions; however, the IFI30 protein levels significantly increased in all MM cell lines treated with IFN-γ (Fig. S2A-C). Previous studies showed that IFN-γ treatment promotes the upregulation of PD-L1 in MM tumor cells [35, 36]. As expected, PD-L1 levels also increased over the time of IFN-γ stimulation (Fig. S2B-C). To identify potential downstream target molecules, *IFI30* was knocked down in four melanoma LNM-derived cell lines (MH-0331-ME, SR-0788-ME, ML-0817-ME, and SG-0035-ME) that showed high-IFI30 protein levels under resting conditions. In all four cell lines, *IFI30* knockdown reduced PD-L1 expression in resting conditions and upon IFN-γ stimulation (Fig. 1O). Similarly, knockdown of IFI30 resulted in down regulation of PD-L1 in MM cell lines treated with IFN-γ, using indirect immunofluorescence assays (Fig. 1P-Q). Further supporting these observations, PD-L1 protein levels had a dose-dependent response to small-interference RNA (siRNA) targeting *IFI30* (Fig. S2D). Then, the subcellular distribution of IFI30 and PD-L1 was determined before and after IFN-γ stimulation using subcellular fractionation of MM cell lines. IFI30 and PD-L1 proteins were mainly localized in the cytosol fraction of MM cell lines with and without IFN-g stimulation (Fig. 1R). In summary, IFI30 and PD-L1 protein levels are upregulated by IFN-γ stimulation. Of note, PD-L1 protein levels showed a dependency on IFI30 protein levels in MM cells under basal as well as IFN-γ conditions, suggesting that IFI30 may have a role in controlling PD-L1 expression.

### IFI30 knockdown increased PD-L1 protein degradation

PD-L1 protein levels and stability are regulated by trafficking from the plasma membrane to recycling endosomes and lysosomes [7, 8]. CKLF-Like MARVEL Transmembrane Domain Containing 6 (CMTM6), and Huntingtin-interacting protein 1-related protein (HIP1R) were identified as critical regulators of PD-L1 transportation [7, 8]. CMTM6 interacts with PD-L1 at the plasma membrane and in recycling endosomes where it protects PD-L1 from lysosomal-mediated degradation [8]. In contrast, HIP1R binds to PD-L1 and targets PD-L1 for lysosomal-mediated degradation [7]. IFI30 resides in late endosomes and lysosomes [37] and reduces the activity of the lysosomal cysteine protease cathepsin L (CTSL) to prevent viral glycoproteins activation [38]. CTSL predominantly exhibits endoprotease activity that is essential for lysosomal protein degradation [39, 40]. IFI30 decreases CTSS protein expression and decreases CTSS and CTSL activity [30]. Therefore, we hypothesized that IFI30 suppresses PD-L1 degradation by inhibiting CTSL.

**Fig. 2:**
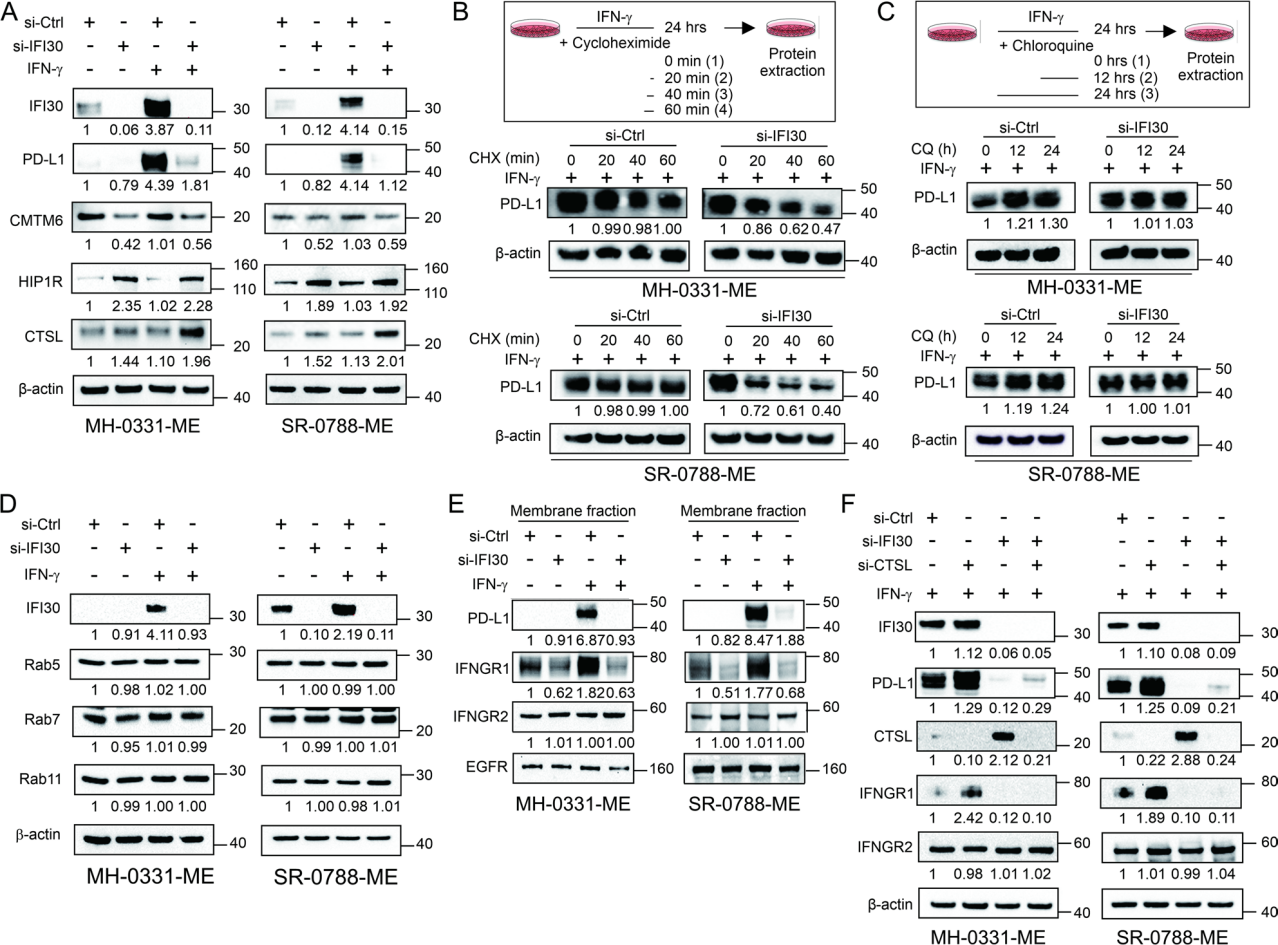
IFI30 knockdown promotes PD-L1 protein degradation. **A** Western blot images show the levels of IFI30, PD-L1, CMTM6, HIP1R, and CTSL levels in MM cell lines (MH-0031-ME and SR-0788-ME) control (si-Ctrl) or IFI30 knockdown (si-IFI30) untreated or treated with IFN-γ. **B** Cycloheximide (CHX) chasing assay and Western blot images show the levels of PD-L1 in melanoma cell lines (MH-0031-ME and SR-0788-ME) control (si-Ctrl) or *IFI30* knockdown (si-*IFI30*) after 0, 20, 40, 60 min of incubation with CHX. **C** Western blot images show the levels of PD-L1 in MM cell lines (MH-0031-ME and SR-0788-ME) control (si-Ctrl) or *IFI30* knockdown (si-*IFI30*) after 0, 12, 24 h of incubation with chloroquine (CQ). **D** Western blot images show IFI30, Rab5, Rab7, and Rab11 levels in MM cell lines (MH-0031-ME and SR-0788-ME) control (si-Ctrl) or *IFI30* knockdown (si-*IFI30*) untreated or treated with IFN-γ. **E** Western blot images show the levels of PD-L1, IFNGR1, IFNGR2 in membrane fractions isolated from MM cell lines (MH-0031-ME and SR-0788-ME) control (si-Ctrl) or *IFI30* knockdown (si-*IFI30*) untreated or treated with IFN-γ. **F** Western blot images show the levels of PD-L1, CTSL, IFNGR1, IFNGR2 in MM cell lines control (si-Ctrl) or *IFI30* knockdown (si-*IFI30*) in absence or presence of si-CTSL during IFN-γ stimulation. β-actin was used as loading control in (A, B, C, D, F). EGFR was used as loading control in (**E**)

Based on the above-described studies, CTSL, CMTM6, and HIP1R protein levels were analyzed in MM cell lines with *IFI30* knockdown. Of note, *IFI30* knockdown significantly decreased CMTM6 protein levels, while it increased HIP1R and CTSL protein levels, both in untreated and IFN-γ treated conditions (Fig. 2A). The CTSL protein levels were upregulated by IFN-γ treatment in a time-dependent manner, while no changes were observed for CMTM6 and HIPR1 (Fig. S2C). In unstimulated MM cells treated with increasing concentration of siRNA targeting *IFI30*, CMTM6 protein levels significantly decreased while the protein levels of HIP1R and CSTL significantly increased (Fig. S2D). These results suggested that IFI30 negatively regulates CTSL and HIP1R protein levels, but they also suggest that CMTM6 protein levels depend on the presence of IFI30. Of note, CSTL and IFI30 protein levels were upregulated in response to IFN-γ stimulation.

Next, we evaluated whether *IFI30* downregulation promotes PD-L1 degradation in cycloheximide (CHX) chase assay as well as chloroquine (CQ) treatment. CHX is an inhibitor of protein synthesis, while CQ blocks lysosomal acidification which is required for activation of lysosomal proteases including by cathepsins. CQ treatment reduces PD-L1 lysosomal-mediated degradation and promotes PD-L1 endosomal recycling [41, 42]. Therefore, MM cell lines were treated with CHX or CQ to set up the optimal incubation time conditions as 60 min and 24 h respectively (Fig. S3A-B). Of note, PD-L1 levels significantly decreased 60 min after CHX treatment in *IFI30* knockdown conditions, while not significant changes were observed in control conditions (Fig. 2B). These results suggest that IFI30 may control PD-L1 protein stability. In contrast, the incubation with CQ prevented PD-L1 degradation in MM cell lines with *IFI30* knockdown (Fig. 2C), suggesting that IFI30 is an important factor controlling PD-L1 lysosomal-mediated degradation. Of note, the levels of the Rab proteins (Rab5, Rab11, Rab7) implicated in the transport of specific vesicles from the membrane to early endosomes to lysosomes were not affected by *IFI30* knockdown (Fig. 2D), suggesting that the changes triggered by IFI30 are not due to alterations in the vesicular transport of PD-L1. In summary, *IFI30* knockdown promotes PD-L1 lysosomal-mediated degradation to decrease PD-L1 protein levels.

To determine whether the membrane levels of PD-L1 depend on the presence of IFI30, MM cell lines with *IFI30* knockdown or control conditions were treated with IFN-γ, lysed, and differentially centrifuged to obtain membrane fractions. In Western blot analysis, the highest PD-L1 protein levels were observed in control MM cell lines treated with IFN-γ, while PD-L1 significantly decreased in MM cell lines with *IFI30* knockdown (Fig. 2E). Of note, only IFNGR1, but not IFN-γ receptor 2 (IFNGR2) or epidermal growth factor receptor (EGFR, loading control), significantly decreased in MM cell lines with *IFI30* knockdown untreated or treated with IFN-γ (Fig. 2E). Supporting the downregulation of PD-L1 in membrane fractions, immunoprecipitation (IP) of Rab11— a marker of recycling endosomes — showed a decreased PD-L1 levels in the IP fraction of *IFI30* knockdown MM cell lines treated with IFN-γ (Fig. S3C). These results suggested that *IFI30* knockdown decreased PD-L1 levels in membrane fractions of IFN-γ treated MM cell lines. OF note, *IFI30* knockdown may also have implications in determining the membrane levels of IFNGR1.

Based on the result showing that CTSL is upregulated by IFN-γ treatment and that *IFI30* downregulation increased the levels of CTSL, we examined the function of CTSL in controlling PD-L1 levels during IFN-γ stimulation. Surprisingly, *CTSL* knockdown increased PD-L1 and IFNGR1 protein levels under basal conditions (Fig. 2F), suggesting that CTSL is responsible for PD-L1 and IFNGR1 protein degradation. In MM cell lines co-transfected with *IFI30* and *CTSL* siRNAs, we observed a partial recovery on PD-L1 levels, but not for IFNGR1. These results suggested that CTSL partially controls PD-L1 lysosomal-mediated degradation. However, *IFI30* knockdown abolished PD-L1 and IFNGR1 protein levels, and CTSL knockdown partially recovered PD-L1 protein levels. These results suggest that *IFI30* knockdown controls PD-L1 protein levels by an upstream mechanism in addition to preventing the CTSL-mediated lysosomal degradation.

### IFI30 knockdown decreased PD-L1 expression at the mRNA level

**Fig. 3:**
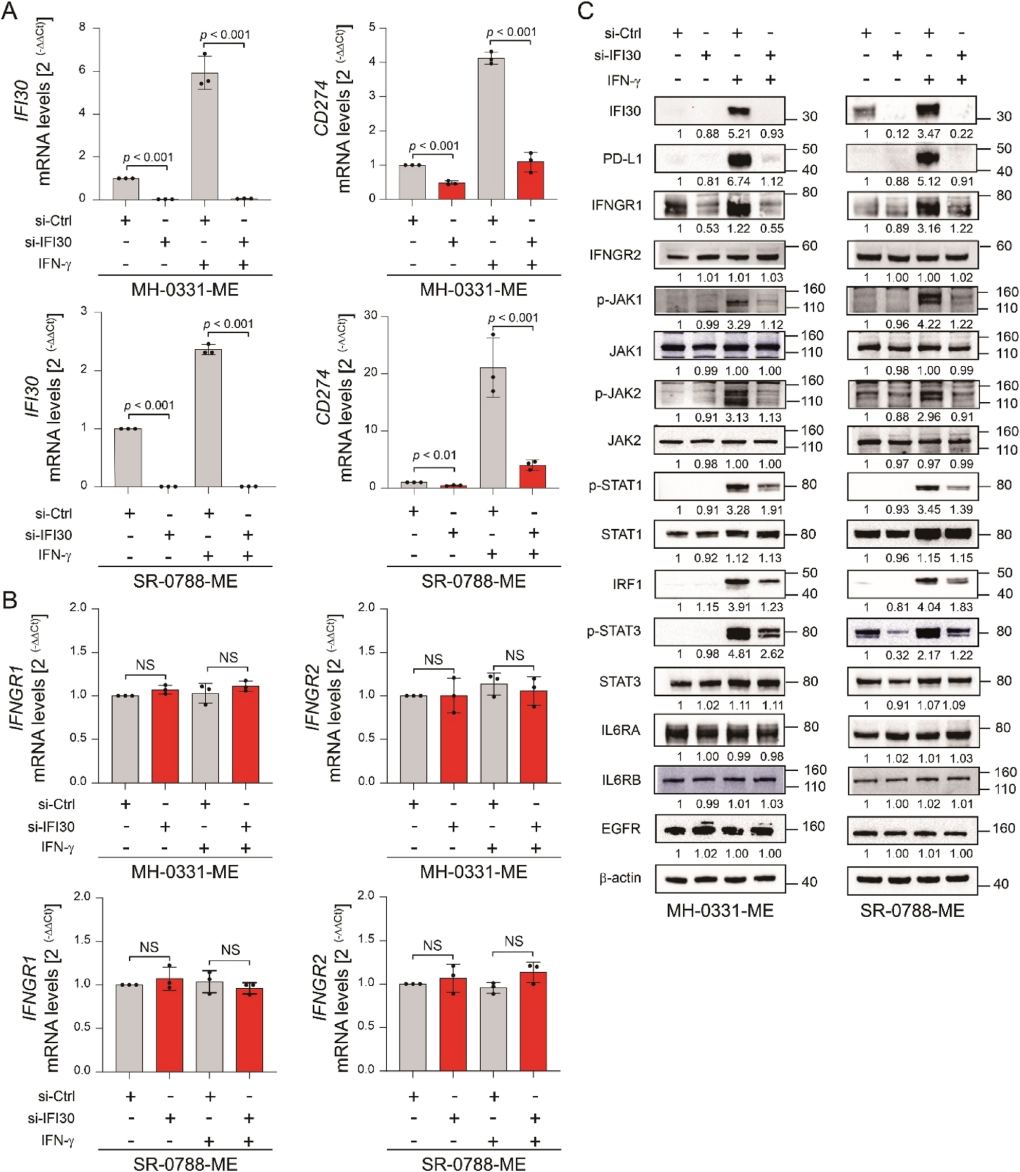
*IFI30* knockdown reduces PD-L1 expression at the mRNA level. **A** Bar plot shows the mRNA levels of *IFI30* and *CD274* in MM cell lines (MH-0031-ME and SR-0788-ME) control (si-Ctrl) or *IFI30* knockdown (si-*IFI30*) untreated or treated with IFN-γ. **B** Bar plot shows the mRNA levels of *IFNGR1 *and *IFNGR2* in MM cell lines (MH-0031-ME and SR-0788-ME) control (si-Ctrl) or *IFI30* knockdown (si-*IFI30*) untreated or treated with IFN-γ. **C** Western blot images show the levels of IFI30, PD-L1, IFNGR1, IFNGR2, pJAK1, JAK1, pJAK2, JAK2, pSTAT1, STAT1, IRF1, pSTAT3, STAT3, IL6RA, IL6RB, and EGFR levels in MM cell lines (MH-0031-ME and SR-0788-ME) control (si-Ctrl) or *IFI30 *knockdown (si-*IFI30*) untreated or treated with IFN-γ. Data represents the mean ± SD. NS: not significant. Significance was determined by One-Way ANOVA in (**A**, **B**)

Based on the above results, we looked for potential connections between IFN-γ pathway activation and PD-L1 transcriptomic regulation in MM cell lines with *IFI30* knockdown using RT-qPCR. *IFI30* knockdown reduced PD-L1 (*CD274*) mRNA levels in MM cell lines in untreated conditions and upon IFN-γ stimulation (Fig. 3A), suggesting a regulatory mechanism driven by IFI30 controlling PD-L1 transcription. Therefore, we hypothesized that *IFI30* knockdown leads to the degradation of specific receptors implicated in PD-L1 transcriptomic regulation, such as IL6 receptor (IL6R) and the IFNGR, which is composed of two subunits: IFNGR1 and IFNGR2. IFNGR1 directly interacts with the IFN-γ ligand, while IFNGR2 is necessary for signal transduction [43]. Upon IFN-γ stimulation, IFNGR phosphorylates and activates Janus kinase 1 (JAK1) and Janus kinase 2 (JAK2) [44, 45]. Phosphorylated-JAK1/2 (pJAK1/2) subsequently phosphorylate signal transducer and activator of transcription 1 (STAT1) and signal transducer and activator of transcription 3 (STAT3). Phosphorylated-STAT1/3 (pSTAT1/3) translocate into the nucleus and promote the transcription of target genes [44]. 

Importantly, upon IFN-γ activation, the IFNGR1 protein is endocytosed, transported to lysosomes, and predominantly degraded in lysosomes [46], while IFNGR2 is not endocytosed [47, 48]. Therefore, we inferred that *IFI30* knockdown promotes the lysosomal-mediated degradation of IFNGR1 and downstream pJAK1/2 and pSTAT1/3 deactivation to reduce PD-L1 mRNA levels in *IFI30* knockdown cell lines. Consistently, qRT-PCR analysis demonstrated that *IFNGR1*, *IFNGR2*, and *CTSL* mRNA levels were not affected by *IFI30* knockdown (Fig. 3B and Fig. S3D-E). Western blot analysis demonstrated that IFNGR1 protein levels were decreased by *IFI30* knockdown condition, while no changes were observed in IL6RA, IL6RB, and IFNGR2 protein levels (Fig. 3C). Consequently, *IFI30* knockdown reduced the phosphorylation levels of pJAK1/2 and pSTAT1/3 or the levels of IRF1 — a downstream target of IFN-γ pathway — in MM cell lines stimulated with IFN-γ (Fig. 3C). In summary, the results suggested that the IFNGR1 degradation in MM cell lines with *IFI30* knockdown prevented IFN-γ pathway activation and consequently, decreased PD-L1 mRNA and protein levels in MM cell lines upon IFN-γ stimulation.

### CTSL-mediated degradation of IFNGR1 reduces the levels of IFI30 and PD-L1 during IFN-γ treatment

**Fig. 4:**
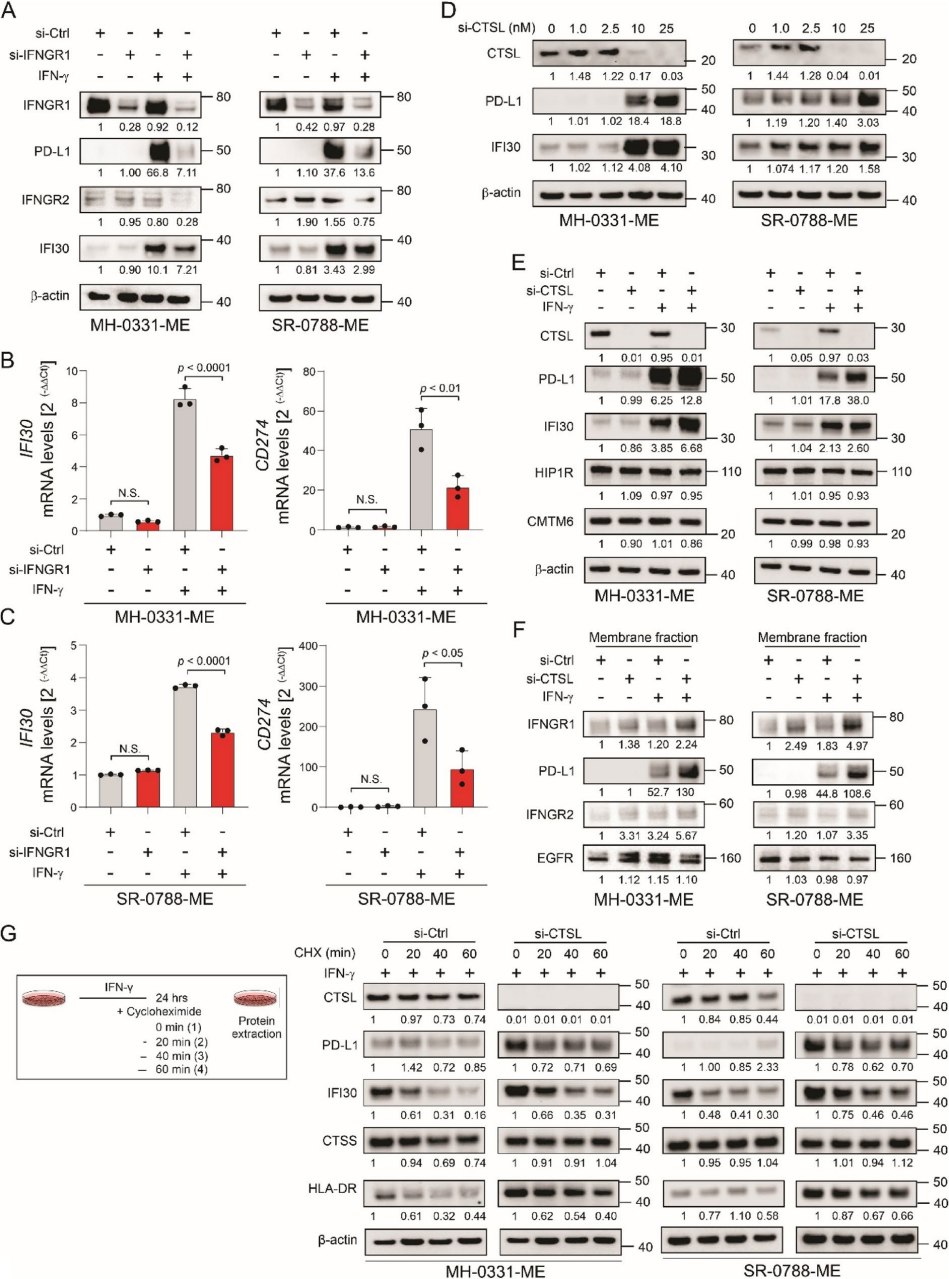
CTSL-mediated degradation of IFNGR1 reduces the levels of IFI30 and PD-L1 during IFN-γ treatment. **A** Western blot images show the levels of IFI30, PD-L1, IFNGR1, and IFNGR2 levels in MM cell lines (MH-0031-ME and SR-0788-ME) control (si-Ctrl) or *IFNGR1* knockdown (si-IFNGR1) untreated or treated with IFN-γ. **B**-**C** Bar plots show the mRNA levels of *IFI30* and *CD274* in MH-0031-ME (**B**) and SR-0788-ME (**C**) si-Ctrl or si-*IFNGR1* untreated or treated with IFN-γ. **D** Western blot images show the changes in CTSL, PD-L1, and IFI30 protein levels using different doses (0, 1, 2.5, 10, and 25 nM) of si-*CTSL* in MM cell lines (MH-0031-ME and SR-0788-ME). **E** Western blot images show the changes in CTSL, PD-L1, IFI30, HIP1R, and CMTM6 protein levels in MM cell lines (MH-0031-ME and SR-0788-ME) control (si-Ctrl) or *CTSL* knockdown (si-*CTSL*) untreated or treated with IFN-γ. **F** Western blot images show the levels of PD-L1, IFNGR1, IFNGR2 in membrane fractions isolated from MM cell lines (MH-0031-ME and SR-0788-ME) si-Ctrl or si-*CTSL* untreated or treated with IFN-γ. β-actin was used as loading control in (**A, E, D**) and EGFR was used as loading control in **F**. **G** Cycloheximide (CHX) chasing assay and Western blot images show the levels of CSTL, PD-L1, IFI30, CTSS, and HLA-DR in melanoma cell lines (MH-0031-ME and SR-0788-ME) control (si-Ctrl) or *CTSL* knockdown (si-*CTSL*) after 0, 20, 40, 60 min of incubation with CHX. Data represents the mean ± SD. NS: not significant. Significance was determined by One-Way ANOVA in (**B**, **C**)

To evaluate our hypothesis, *IFNGR1* was knocked down in MM cell lines. PD-L1 and IFI30 protein levels were significantly decreased in MM cell lines with *IFNGR1* knockdown that were stimulated with IFN-γ (Fig. 4A). Consistent results were obtained in RT-qPCR analysis (Fig. 4B-C), suggesting that both PD-L1 and IFI30 are downstream targets of IFNGR1. Then, the focus was on demonstrating the importance of CTSL in targeting PD-L1 and IFI30 for degradation. The levels of *CTSL* and *IFNGR1* were further assessed in the TCGA-SKCM in PRM and MM tumor samples. However, no significant differences were observed (Fig. S3F-G). Of note, in silico the mRNA or protein levels of *CTSL* positively correlated with *IFI30* mRNA or protein levels (Fig. S4A-B and S5A, E, I) or with *CD274* mRNA levels (Fig. S5B, F). In vitro, *CTSL* knockdown significantly increased PD-L1 and IFI30 protein levels in a dose dependent manner under resting or IFN-γ stimulated conditions (Fig. 4D-E). Of note, CMTM6 and HIP1R are not regulated by CSTL (Fig. 4E). *CTSL* knockdown significantly increased the protein levels of PD-L1 and IFNGR1 in membrane fractions obtained from MM cell lines upon IFN-γ stimulation (Fig. 4F). In CHX chase assays, *CTSL* knockdown significantly increased the basal levels of PD-L1 and IFI30 (Fig. 4G) and partially prevented PD-L1 and IFI30 protein degradation. These results suggest that CSTL is a critical factor controlling IFNGR1 levels and, hence IFN-γ pathway activation, which negatively controls IFI30 as well as PD-L1 protein/mRNA levels in MM cell lines.

### IFI30 positively regulates cathepsin-S (CTSS) levels by regulating CTSL-mediated degradation of IFNGR1 during IFN-γ treatment

HLA-DR are the primary antigen-presenting MHC-II molecules expressed in melanoma tumors as a consequence of the local levels of IFN-γ secreted by CD4^+^ T helper 1 cells and CD8^+^ cytotoxic T cells [15, 49]. Previous studies showed that melanoma patients whose tumors had ≥ 5% of HLA-DR positivity had significantly better response to ICI, and improved PFS and OS[13, 15, 49]. Moreover, IFI30 is required for the presentation of a melanoma antigens, and the subsequent generation of a CD4^+^ T cell response [26]. CTSS degrades the invariant chain and MHC-II acquires antigenic peptides, which accelerates the onset and intensity of CD4^+^ T cell responses. The levels of *CTSS* were assessed in the TCGA-SKCM in PRM and MM tumor samples. The results showed a significant upregulation in *CTSS* mRNA levels in MM tissue samples (Fig. S3H). Besides, the mRNA levels of *CTSS* positively correlated with *IFI30* mRNA or protein levels (Fig. S4A-B and S5C, G, J) or with *CD274* mRNA levels (Fig. S5D, H). As expected, CTSS and HLA-DR protein levels increased in MM cell lines stimulated with IFN-γ (Fig. S2C). Supporting these results, the mRNA levels of *IFI30* in deconvoluted melanoma cells positively correlated with almost all MHC-II, including *HLA-DRA* mRNA levels (Fig. S4C, Fig. S6A). Of note, *IFI30* knockdown decreased CTSS and HLA-DR protein levels in MM cell lines in a dose dependent manner (Fig. S2D and S6B). *CTSL* knockdown in MM cell lines did not significantly increase the protein levels of CTSS under resting conditions or IFN-γ stimulated conditions; however, the protein levels of HLA-DR significantly increased in MM cell lines stimulated with IFN-γ (Fig. S6C). *IFNGR1* knockdown significantly decreased CTSS or HLA-DR protein levels under IFN-γ stimulated conditions, but it has no effect on resting conditions (Fig. S6D). These results are consistent with the upregulation of CTSS and HLA-DR mediated by IFN-γ stimulated conditions (Fig. S2C). In CHX analysis, *CTSL* knockdown did not affect the basal protein levels of CTSS or the degradation of CTSS protein levels (Fig. 4G). However, *CTSL* knockdown significantly increased the basal levels of HLA-DR, but it did not alter the rate of the HLA-DR protein degradation in MM cell lines stimulated by IFN-γ (Fig. 4G). These results suggested that IFI30 controls CTSS and HLA-DR levels by modulating lysosomal degradation mediated by CTSL in MM.

### High-IFI30 levels in tumor samples obtained from MM are associated with improved immunotherapy responses

**Fig. 5:**
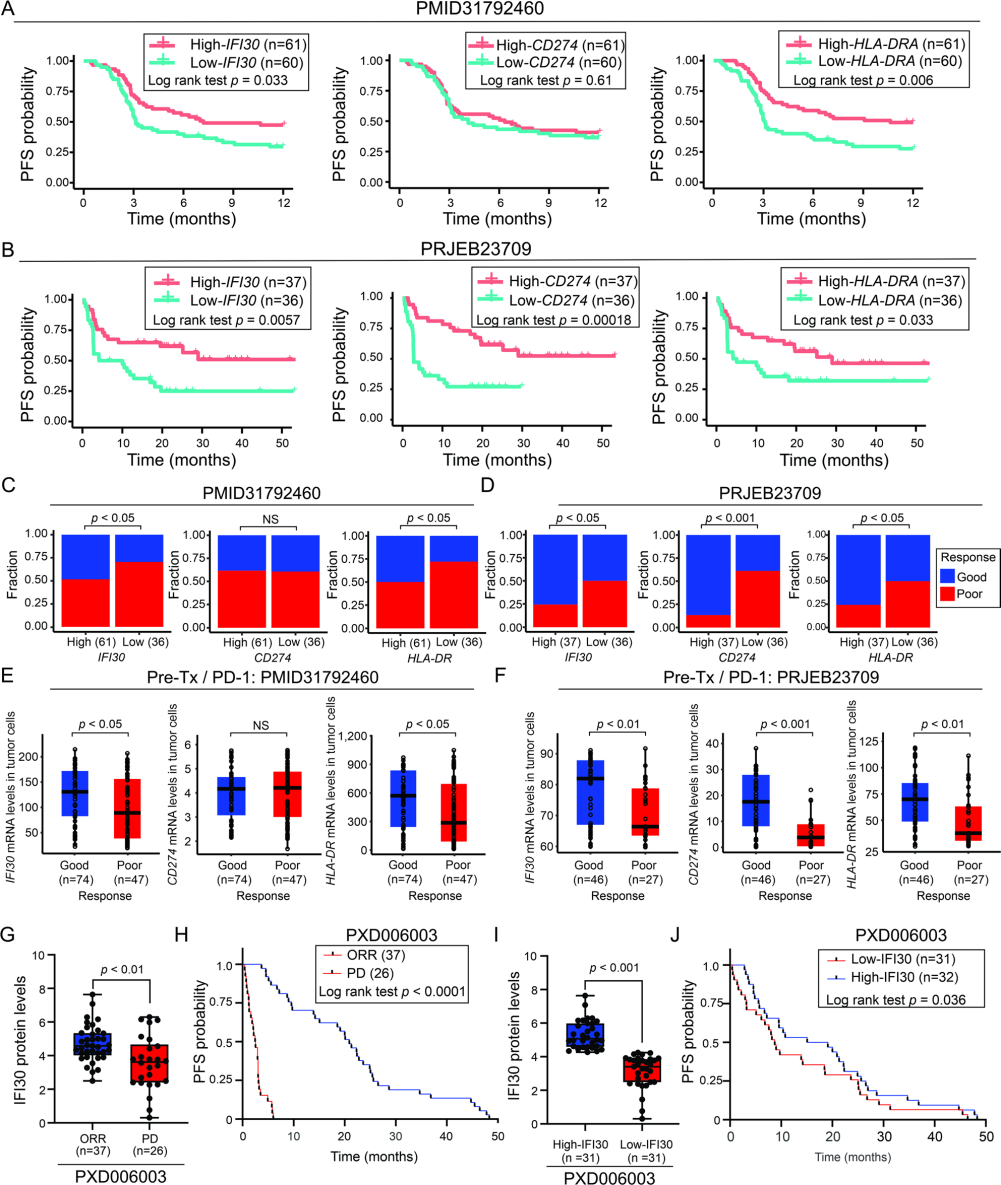
High-*IFI30* levels are associated with better ICI treatment responses. **A**-**B** Kaplan-Meier curves show the progression-free survival (PFS) probability comparing MM patients with high- vs. low-*IFI30*, or high- vs. low-C*D274*, or high- vs. low-*HLA-DRA* mRNA levels from the PMID31792460 (**A**) or PRJEB23709 (**B**) datasets. **C**-**D** Stacked plots show the fraction of MM patients with low- vs. high-*IFI30*, or low- vs. high-*CD274*, or low- vs. high-*HLA-DRA* in pre-treatment samples of PMID31792460 (**C**) or PRJEB23709 (**D**) datasets. **E**-**F** Box plots show IFI30, CD274, and HLA-DRA mRNA levels in pre-treatment samples from MM patients with good and poor response to ICI treatment of PMID31792460 (**E**) or PRJEB23709 (**F**) datasets. **G** Bar graphs show the IFI30 protein levels are significantly higher in patients with ORR compared to those with PD in ICI treatment. **H** Kaplan-Meier curve shows the PFS in patients with objective rate response (ORR) versus progressive (PD). **I** Bar graphs show IFI30 protein levels in MM patients classified into high-IFI30 and low-IFI30 groups. **J** Kaplan-Meier curves show PFS in MM patients classified based on high-IFI30 and low-IFI30 groups. Significance was determined by Wilcoxon U-test in (**C**, **D**, **E**, **F**, **G**, **H**). Log-rank test in (**A**, **B**, **G**, **H**). NS, non-significant

A previous study demonstrated that IFI30 protein levels in melanoma cells were associated with response to ICI [13]. To further validate these findings in silico analysis were performed using two larger publicly available datasets: PMID31792460 and PRJEB23709 [24, 50]. PMID31792460 contains RNA-Seq data for 121 pre-treatment tumor tissue samples obtained from MM patients (stage III/IV) treated with ICIs (anti-PD-1). In this study, MM patients were divided into patients with good response (complete response (CR)/partial response (PR)/stable disease (SD)/mixed response (MR) with PFS > 1 year) and poor response (progressive disease (PD)/SD/PR/MR with PFS ≤ 1 year) groups. PRJEB23709 dataset contains RNA-Seq data for 73 pre-treatment tumor tissue samples obtained from MM patients treated with ICIs (anti-PD-1 ± anti-CTLA-4). Patients were stratified into good response (CR/PR/SD with PFS > 6 months) and poor response (PD/SD with PFS ≤ 6 months) groups. In both datasets, high-*IFI30* or high-*HLA-DRA* levels —defined based on median cutoff values— were associated with improved PFS in MM patients receiving ICIs (Fig. 5A-B and S4A-B). High levels of PD-L1 (*CD274*) showed improved PFS only in the PRJEB23709 dataset, but not in the PMID31792460 dataset (Fig. 5A-B and S4A-B). A significantly higher number of patients having a good response were observed in MM patients stratified based on high-*IFI30* and low-*IFI30* groups in both PMID31792460 and PRJEB23709 datasets (Fig. 5C-D and S4A-B). Similar results were observed in MM patients stratified based on high-*HLA-DRA* and low-*HLA-DRA* groups in both PMID31792460 and PRJEB23709 datasets (Fig. 5C-D and S4A-B). Consistently, MM patients with good response (Complete response (CR)/partial response (PR)/Stable disease (SD) PFS > 1 year) showed significantly higher levels of *IFI30* or *HLA-DRA* (Fig. 5E-F and S4A-B). Finally, high-*CTSS* mRNA levels were associated with improved PFS/OS in MM patients receiving ICIs, but not significant differences were observed in PFS/OS for high-*CTSL* groups (Fig. S7A-H and S4A-B). These results suggest that *IFI30*, *HLA-DRA*, and *CTSS* mRNA levels may represent potential biomarkers associated with ICI responses.

Furthermore, IFI30 protein levels were evaluated in MM patients who had objective rate response (ORR) compared to MM patients with PD to ICI treatment using the PXD006003 public dataset [51]. PXD006003 dataset contains protein for 63 pre-treatment tumor tissue samples obtained from MM patients treated with ICIs (anti-PD-1). In this study, tissues samples from patients with MM were analyzed before patients received ICIs (anti-PD-1). Of note, higher IFI30 protein levels were observed in patients with ORR than PD group (Fig. 5G). As expected, MM patients with ORR showed a significantly better prognosis than MM patients with PD (Fig. 5H). Finally, MM patients were divided based on median protein levels of IFI30 into high- and low-IFI30 groups (Fig. 5E). Concordantly, MM patients with tumors having high-IFI30 levels showed a significantly better PFS (Fig. 5J). In summary, high-IFI30 mRNA/protein levels were associated with improved outcomes in MM patients. Consistent with previously reported data, high-IFI30 mRNA/protein levels were associated with a better response to ICI.

### High-IFI30 levels in melanoma cells are associated with increased immune cells infiltration and better response to PD-1 inhibitors

**Fig. 6:**
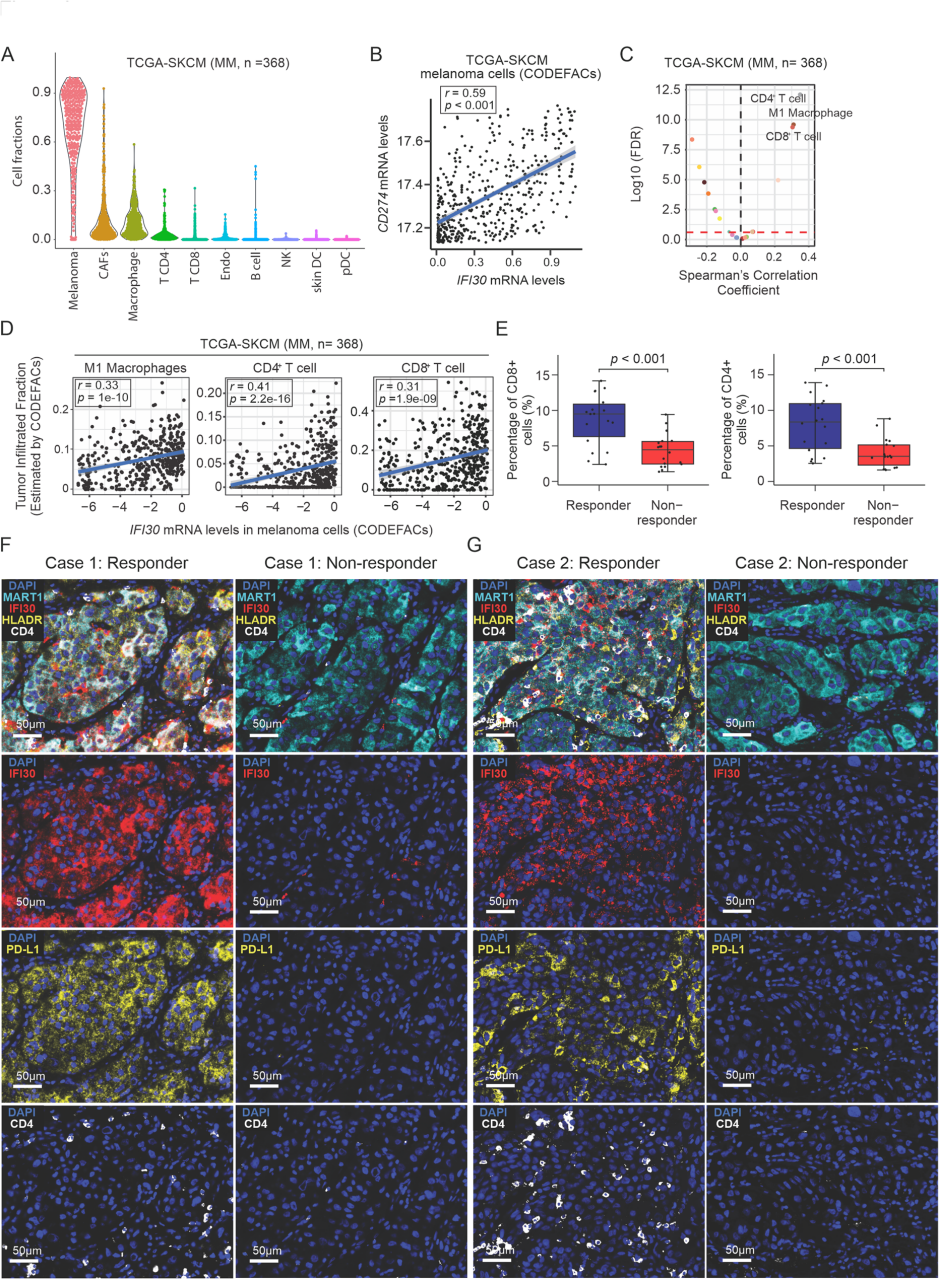
High-IFI30 levels in malignant melanoma cells correlate with immune cell infiltration and better ICI treatment response. **A** Violin plot shows the cell fractions after CODEFACS deconvolution analysis of TCGA-SKCM dataset. **B** Scatter plot shows the correlation of *IFI30* and *PD-L1* mRNA levels in melanoma cells after CODEFACS deconvolution analysis of TCGA-SKCM dataset. **C** Volcano plot shows the correlation values between *IFI30* and immune cell infiltration vs. Log_10_ false discovery rate (FDR) using the TCGA-SKCM dataset. **D** Correlation plot shows the correlation between *IFI30* mRNA levels and M1 macrophages, or CD4^+^ T cells, or CD8^+^ T cells. Correlation values were determined by Spearman’s correlation test (**B-D**). **E** Quantification of the percentage of CD8^+^ and CD4^+^ cells in tissue samples from responder and non-responder MM patients. **F** Representative mIF images of FFPE MM tissue samples stained using Opal multiplex staining panel (1) DAPI (blue); Mart-1 (light blue); IFI30 (red); PD-L1 (yellow); and CD8 (white). Scale bar = 50 μm. **G** Representative mIF images of FFPE melanoma LNM tissue samples stained using Opal multiplex staining panel (2) DAPI (blue); Mart-1 (light blue); IFI30 (red); PD-L1 (yellow); and CD4 (white). Scale bar = 50 μm

CODEFACS deconvolution algorithm was applied to RNA-Seq data obtained from The Cancer Genome Atlas – Skin Cutaneous Melanoma (TCGA-SKCM, *n* = 368) to have a comprehensive analysis on *IFI30* mRNA levels in melanoma cells (Fig. 6A and S4C). Supporting previous results, *IFI30* and *CD274* mRNA levels were significantly correlated in malignant melanoma cells (Fig. 6B and S4C). In addition, the *IFI30* mRNA levels in melanoma cells showed a significantly positive correlation with the infiltration levels of M1 macrophages, CD8^+^ and CD4^+^ T cells in MM tumors (Fig. 6C-D and S4C). Of note, significantly higher levels of CD8^+^ and CD4^+^ T cells were observed in MM patients of the responder group (Fig. 6E). To validate the correlation between *IFI30* mRNA levels in melanoma cells and CD4^+^ or CD8^+^ T cells infiltration levels, melanoma LNM tissue samples were assessed by mIF using two defined antibody panels [Panel 1: (HMB45/MART1, PD-L1, CD8, IFI30) and panel 2: (HMB45/MART1, PD-L1, CD4, IFI30)]. All MM patients were divided into responder (CR/PR) and non-responder groups (SD/PD) based on the response to ICIs (anti-PD-1 ± anti-CTLA-4). Higher IFI30 protein levels were observed in MM tissues samples from the responder group (Fig. 6F-G). In summary, the levels of IFI30 were associated with increased PD-L1 levels as well as increased immune infiltration in LNM obtained from MM patients that responded to ICI treatment.

## Discussion

**Fig. 7:**
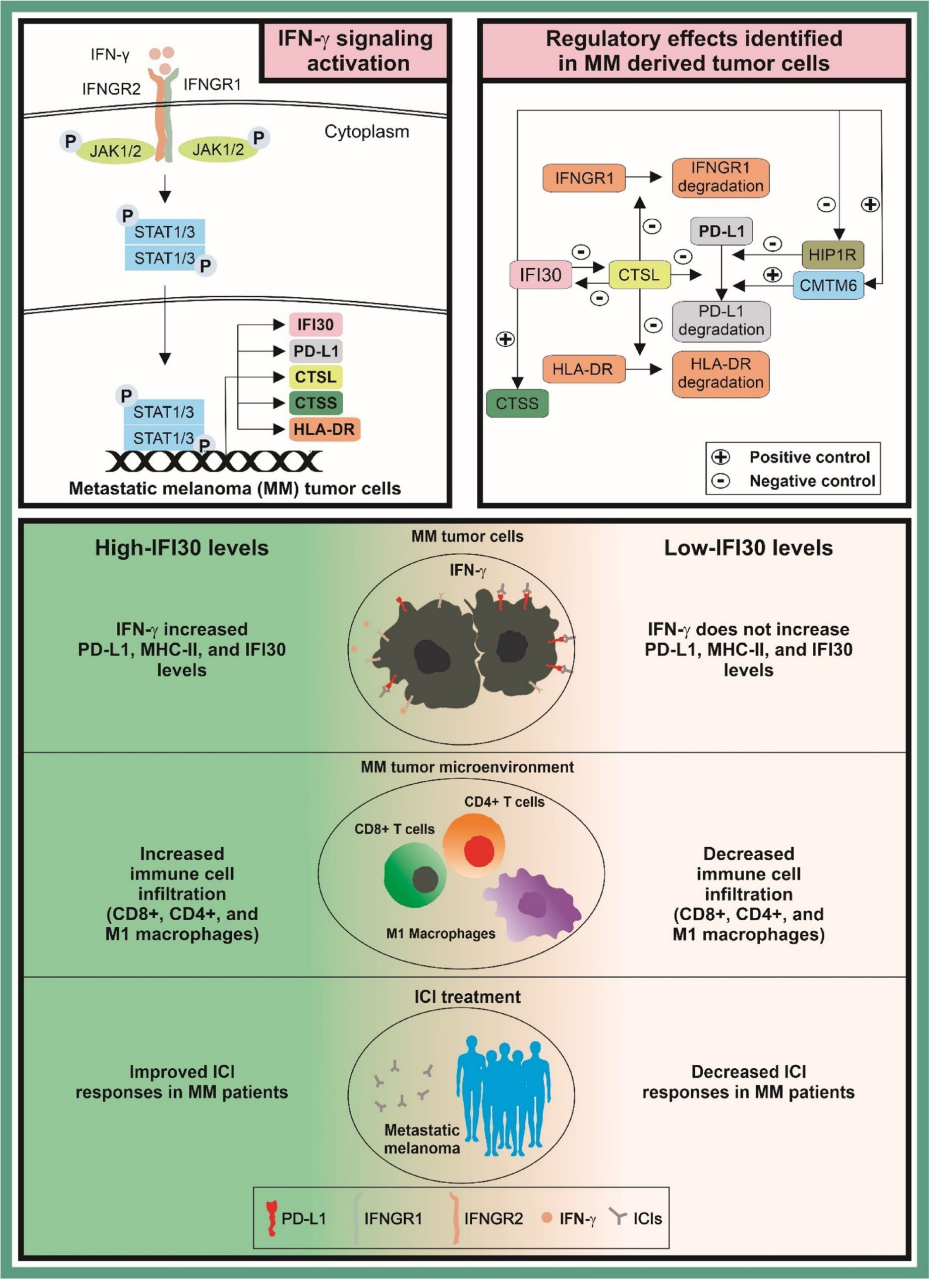
Schematic representation of the molecular mechanism driven by Interferon-gamma induced factor 30 (IFI30) in metastatic melanoma (MM). *Top left*. Downstream targets activated upon IFN-γ signaling activation, including IFI30, PD-L1, and MHC-II molecules. *Top right*. Shown are also the regulatory effects observed in this study in MM tumor cells using in-vitro functional assays combined with small interference RNA. *Bottom*. High-IFI30 levels were associated with higher CD8^+^ and CD4^+^ T cells as well as M1-macrophages infiltrating levels in the tumor immune microenvironment of MM tumors. Consequently, high-IFI30 levels were associated with better response to ICIs

The IFN-γ pathway is critical in controlling the tumor immune microenvironment of MM tumors and the response to ICIs,20 however, there is a still a poor understanding of the multiple factors activated upon IFN-γ stimulation in MM tumors, as well as their molecular function. In this study, we characterized the role of IFI30 as a downstream target of IFN-γ pathway activation. The results of this study show for the first time that IFI30 suppressed IFNGR1 lysosomal-mediated degradation by blocking the activity of CTSL (Fig. 7). Mechanistically, IFI30 knockdown reduced IFNGR1 protein levels to deactivate IFN-γ pathway upon IFN-γ stimulation. Consequently, IFNGR1 downregulation significantly reduced the mRNA and protein levels of PD-L1 and HLA-DR upon IFN-γ treatment. At the protein level, IFI30 knockdown did not block PD-L1 and HLA-DR lysosomal degradation mediated by CTSL. In clinical samples, the levels of PD-L1 and HLA-DR levels were significantly associated with improved PFS and OS in MM receiving ICI treatment, as well as better responses to ICI treatment. In deconvolution assays high IFI30 levels were consistently associated with increased levels of CD4 + or CD8 + T cells (Fig. 7).

The regulatory mechanisms of PD-L1 levels have been investigated based on the importance of ICIs targeting PD-L1 as well as the negative impact that PD-L1 has in immune surveillance [7]. The degradation and recycling of plasma membrane proteins is mediated by endolysosomal pathway. In the endolysosomal pathway, PD-L1 at the plasma membrane is first monoubiquitinated and internalized into the cell via clathrin-mediated endocytosis to early endosomes. Then, PD-L1 is sorted from early endosomes to recycling endosomes for recirculation to the cell surface, or to late endosomes/lysosomes through multivesicular bodies (MVB) for degradation [52, 53]. Previous studies have identified CMTM6 and HIP1R as key regulators of PD-L1 transport and degradation mediated by the endolysosomal pathway [7, 8]. While CMTM6 works as a positive regulator of PD-L1 protein recycling, HIP1R is a negative regulator that promotes PD-L1 protein degradation [7, 8]. Surprisingly, *IFI30* knockdown significantly reduced the levels of CMTM6, while *IFI30* knockdown enhanced the protein levels of HIP1R independent of IFN-γ stimulation. However, the effect of IFI30 on HIP1R and CMTM6 does not depend on the levels of CTSL, as observed in *CTSL* knockdown experiments. Nonetheless, these findings will require further investigation to determine the role of IFI30 in modulating CMTM6 and HIP1R protein levels in MM cell lines.

CTSL predominantly exhibits endoprotease activity that is essential for protein degradation. Our results show that *IFI30* knockdown significantly increased the levels of CTSL in a time-dependent manner on IFN-γ incubation and in dose-dependent manner on siRNA targeting IFI30. Conversely, *CTSL* knockdown increased IFI30 in untreated as well as after IFN-γ stimulation. These results suggest an exquisite mutual regulation between IFI30 and CTSL that is responsible for CTSL-mediated PD-L1 degradation or PD-L1 recycling to the plasma membrane.

CTSS is a potent protease that belongs to the lysosomal cysteine protease family, suggested to have overlapping functions with CTSL. CTSS degrades the invariant chain, an MHC-II-associated protein that inhibits peptide binding to MHC-II, accelerating the onset of CD4^+^ T cell responses [26, 54]. Similarly, IFI30 reduces disulfide bonds in peptide antigens in the endocytic pathway, and therefore, IFI30 promotes peptide processing and even peptide-MHC-II complex formation, which activates MHC-II antigen-presentation [26, 54]. In B cells, IFI30 and CTSS have been reported to co-localize within lysosomes [30]. In MM, IFI30 is critical for MHC-II-restricted presentation of multiple melanoma antigens and in accelerating the onset of CD4^+^ T cells [12, 27, 32]. In this study, *IFI30* downregulation suppressed CTSS and HLA-DR protein levels, which may directly impact antigen-presentation. CTSS is stimulated by IFN-γ treatment and *CTSL* knockdown increased the protein levels of CTSS and HLA-DR upon IFN-γ stimulation. These results suggested that IFI30 may be a positive regulator of CTSS and HLA-DR by controlling the levels of CTSL in MM tumors. Supporting these findings, high levels of IFI30, CTSS, or HLA-DRA were significantly associated with improved PFS and OS, and better responses to ICIs in MM patients.

Previous studies reported that pretreatment tissue samples from MM patients who responded to PD-1 inhibitors had higher PD-L1 expression in tumor cells and increased infiltration of CD8^+^ T cells [55]. Therefore, infiltration of CD8^+^ T cells is a critical indicator of tumor regression in MM patients receiving PD-1 inhibitors [55]. CD8^+^ T cells are activated to attack tumor cells by the IFN-γ produced and released by CD4^+^ T cells. Furthermore, activated CD8^+^ T cells also produce IFN-γ, which further enhances the immune response against tumor cells [56]. Supporting this observation, high-IFI30 mRNA/protein levels were associated with increased immune cells infiltration (CD4^+^/CD8^+^) and better responses to PD-1 inhibitors. Therefore, the levels of IFI30 may serve as a potential biomarker to stratify MM patients who will have better outcomes and responses to ICIs.

## Conclusions

IFI30 plays a significant role in controlling the levels of IFNGR1, which are critical to maintaining the levels of PD-L1 and downstream molecules stimulated by IFN-γ pathway activation (Fig. 7). Additionally, during IFN-γ stimulation IFI30 promotes the upregulation of PD-L1 and MHC-II molecules by blocking CTSL-mediated protein degradation. Therefore, IFI30 plays a key role in controlling IFN-γ signaling pathway and immune responses against MM tumor cells, suggesting its potential as a biomarker to guide ICI treatment decisions in MM patients.

## Supplementary Information

Supplementary Material 1.

Supplementary Material 2.

Supplementary Material 3.

## Data Availability

The melanoma scRNA-Seq dataset used as a reference signature matrix for CODEFACS deconvolution is publicly available (PMID34983745) [57]. The TCGA-SKCM dataset is available from data generated by the TCGA Research Network (https://www.cancer.gov/tcga). The PRJEB23709 [24] and PMID31792460 [50] were graciously provided by the authors. The PXD006003 [51] dataset is available on the PRIDE repository. The RNA-Seq data from paired melanoma cell lines control or treated with IFN-γ is available at GSE154996. The HTG-AI dataset for melanoma lymph node metastasis tissue samples supporting the conclusions of this article is available at GSE261484.
