# Supplementary material for: Interferon-gamma-inducible protein 30 prevents IFN-γ-receptor 1 degradation to maintain PD-L1 and MHC-II levels in metastatic melanoma
Source: Cell Commun Signal. 2026 Feb 12;24:182. doi: 10.1186/s12964-026-02710-9 (PMC12998310; doi:10.1186/s12964-026-02710-9)
Supplement: Supplementary file 2 — Supplementary Material 2. [file 12964_2026_2710_MOESM2_ESM.docx]

**Interferon-gamma-inducible protein 30 prevents IFN-γ-receptor 1 degradation to maintain PD-L1 and MHC-II levels in metastatic melanoma**

Shodai Mizuno^1#^, Yuka Mizuno^1#^, Kodai Abe^1^, Anne M. Macy^2,3^, Kelly Chong^1^, Yuta Kobayashi^1^, Karen T. Hastings^2,3,4^, Dave S. B. Hoon^1, 3^ and Matias A. Bustos^1^

**Affiliations of authors:**

^1^Department of Translational Molecular Medicine, Saint John’s Cancer Institute (SJCI), Providence Saint John’s Health Center (SJHC), Santa Monica, CA 90404, USA.

^2^Phoenix Veterans Affairs Health Care System, Phoenix, AZ, USA.

^3^Department of Dermatology, College of Medicine - Phoenix, University of Arizona, Phoenix, AZ, USA.

^4^University of Arizona Cancer Center, University of Arizona, Tucson, AZ, USA.

^5^Department of Genome Sequencing Center, SJCI, Providence SJHC, Santa Monica, CA 90404, USA.

# These authors contributed equally.

**Address correspondence and reprint requests to:**

Matias A. Bustos; Dept. of Translational Molecular Medicine, SJCI, Providence SJHC, 2200 Santa Monica Blvd, Santa Monica, CA, 90404, USA.

Email: matias.bustos@providence.org

**Conflict of Interest**

The authors declare no conflicts of interest.

**Supplementary Information**

**Samples Processing for HTG-AI assay**

The analysis was performed in 5 μm formalin-fixed paraffin-embedded (FFPE) sections obtained from LNM positive (LNM (+); n = 17) and LNM negative (LNM (-); n = 8) of patients who received surgery at SJHC. The tumor areas were marked by referring to the H&E staining of the consecutive slide, with the accordance of a certificated pathologist. The estimated tissue areas were measured using Image J software. Then, the optimal amounts of tissue for each HTG EdgeSeq AI assay were scraped and placed into microfuge tubes. The calculated volume of lysis buffer for each collected sample was added, and the samples were then overlaid with 500 μL of HTG denaturation oil. After centrifugation, the samples were incubated at 95 °C for 15–20 min to denature protein structures and remove paraffin wax from the FFPE tissue sections. Samples were cooled down for 10 min at room temperature (RT), and HTG provided Proteinase K was pipetted into the aqueous (lysis, non-oil) phase of the samples at a volume 1/20th of the total lysis buffer. Finally, the samples were incubated at 50 °C for 3 h, with the aqueous phase being mixed by pipetting every 30 min. Subsequently, 25 μL of the sample lysate was loaded onto the HTG EdgeSeq instrument for probe-capture for 20 h.

**Library preparation for HTG-AI assay**

Probe-captured FFPE samples for HTG-AI assay were amplified and indexed via polymerase chain reaction (PCR) using the master mix (OneTaq HotStart 2X MasterMix in GC Buffer (New England Biolabs Inc., Ipswich, MA, USA), and indexing primers. The PCR reaction conditions were as follows: (1) 95 °C for 4 min, (2) 95 °C for 15 s, (3) 56 °C for 45 s, (4) 68 °C for 45 s, (5) repeating steps 2–4 for a total of 19 cycles, (6) 68 °C for 10 min, and (7) holding at 4 °C. Following PCR, library cleanup was performed with a mixture of clean up buffer (39% of 5M NaCl, 31.25% of 40% PEG 8000, 29.75% of molecular-grade water) and AMPure XP beads, by combining it with the PCR-amplified sample at 5:2 ratio, respectively. After 5 min of incubation, the samples were placed on the magnetic stand, then washed twice with 80% ethanol, followed by a 5-min air drying period. The elution was performed using 40 μL of 10mM Tris-HCl, pH 8.0.

**NGS library quality check for HTG-AI assay**

All libraries were quantitated using the KAPA Library Quant Kit (Illumina Inc., San Diego, CA, USA) and the Universal qPCR Mix Kit (Roche, Basel, Switzerland) in accordance with the manufacturer’s recommendations. Quality checks (QC) for library size were performed on the Agilent Technologies TapeStation 2200 instrument using the High Sensitivity D1000 ScreenTape and High Sensitivity D1000 reagents (Agilent Technologies Inc., Santa Clara, CA, USA). The expected peak size ranged between 150-170 base pairs. Samples that did not indicate proper library formation were excluded from sequencing and the library preparation process was repeated.

**NGS library normalization and pooling for HTG-AI assay**

Quantitated libraries were diluted, normalized, and pooled based on the raw quantity (pM) generated by the HTG EdgeSeq RUO Library calculator software version 2.0.0. Then, libraries were denatured in 0.2 N NaOH for 8 min at RT. NaOH was hydrolyzed with 200 mM Tris pH 7.4, and the denatured pool was then diluted down to 4 pM using the Hyb buffer supplied in the NextSeq 550 High Output Kit v2.5 (Illumina Inc., San Diego, CA, USA). To introduce sequencing diversity and a positive sequencing control, 4 pM of 12.5 pM PhiX Control v3 (Illumina Inc., San Diego, CA, USA) was spiked into the diluted and denatured 4 pM sample pool. The final pool consisted of 95% sample libraries and 5% PhiX control by volume. The pooled library was then denatured at 98 °C for 4 min and immediately placed on ice for at least 5 min before loaded onto the Illumina NextSeq 550 instrument, following the Illumina instrument sequencing protocol.

**NGS profiling of the Libraries for HTG-AI assay**

Sequencing on Illumina platforms was conducted according to the HTG instructions, with a read length of 1 × 50 base pairs. The raw sequencing data was transformed into FASTQ files using Illumina BaseSpace BCL to FASTQ software version 2.2.0 and Illumina Local Run Manager Software version 2.0.0. FASTQ files were analyzed with HTG EdgeSeq Parser software version v5.1.724.4793 to generate raw counts for a total of 2,002 genes related to tumor-immune interaction and 18 control genes.

**Targeted RNA-Seq data analysis**

The raw read counts were normalized using: <https://bioconductor.org/packages/release/bioc/vignettes/DESeq2/inst/doc/DESeq2.html>. Differential Expression Outputs including mean normalized values in each group, fold change (FC), raw p-value and adjusted p-value (p-value for each probe after adjustment using the Benjamini and Hochberg method for controlling the false discovery rate) between groups were calculated. PCA was used to determine sample clustering. In all HTG-AI data comparisons, a Log2|FC | ≥ 1 and adjusted p value < 0.05 as significant difference between groups was considered.

**Multiplex immunofluorescence**

Sixteen LNM FFPE tissue sections were obtained from MM patients before receiving ICI treatment. For comparison, MM patients were grouped based on treatment response into responders (CR/PR/SD, n = 8) and non-responders (PD, n = 8). Multiplex immunofluorescence (mIF) was performed on the tissue sections using Opal 7-color manual IHC Kit (NEL 811001KT, Akoya Biosciences). Staining was performed as previously described ^1^. The antibodies utilized, the selected panels, and the corresponding fluorophores are summarized in **Table S3**. The clinical information of the MM patients included in the mIF staining is shown in **Table S2**. mIF-stained slides were imaged using the Mantra Multispectral Imaging System (v1.0, Akoya Biosciences) as previously described ^1^. For quantification, double positive score was calculated using the InForm software (Akoya Biosciences) according to manufacturers’ instructions and as previously described ^1, 2^. The optical signal threshold to classify the double positive score was set to 1.330 for IFI30, 1.330 for PD-L1, 0.600 for Mart-1, 1.250 for CD4, 1.500 for CD8, and 1.280 for M1 macrophages, respectively. For double positive mIF scores, the protein positive areas were automatically segmented, and nuclear/cytoplasm compartments were distinguished automatically by detecting the intensity of nuclear staining (DAPI). In each slide, three to six photographs per sample were captured at 20X magnification. The median scores of the areas evaluated in responder and non-responder groups were compared for statistical significance.

**RNA isolation and RT-qPCR**

Total RNA from MM cell lines was extracted by the Direct-zol RNA miniprep kit (Zymo Research, Irvine, CA) according to the manufacturer’s instructions. Reverse transcription-quantitative polymerase chain reaction (RT-qPCR) was then performed for 1 ng of total RNA. The mRNA levels were quantified using RT-qPCR by a 3-step cycling protocol as previously described ^3^. Primer sets (Integrated DNA Technologies, IA) used in RT-qPCR are shown in **Table S5**.

**Transfection assays**

Knockdown experiments were performed as previously described ^2, 4^. All the siRNA utilized in the experiments are summarized in **Table S3**. Knockdown efficiency in each condition was confirmed by qRT-PCR and Western blot.

**Nuclear extraction**

Nuclear and cytoplasmic fractions were isolated from the MH-0331-ME and SR-0788-ME cell lines using the Nuclear Extract Kit (Active Motif, CA, USA) as previously described ^4^. The nuclear and cytoplasmic fractions were collected and analyzed by Western blot.

**Membrane extraction**

Membrane fractions were isolated from the MH-0331-ME and SR-0788-ME cell lines using the Nuclear Extract Kit (Active Motif, CA,USA). Briefly, MM cells were cultured in 60 mm dishes and harvested 1.5 mL cold PBS/phosphatase inhibitor. MM cells were centrifuged and the whole-cell pellet was gently suspended in 250 μL hypotonic buffer and incubated for 15 min on ice. Then, 12.5 μL of detergent was added to induce cell lysis. After cell lysis, the cytoplasmic fraction (supernatant) was separated from the nuclear fraction (pellet) by centrifugation (2 minutes at 14,000 × *g*). Then, the supernatant was centrifuged (1 hour at 100,000 × *g*) and membrane fractions (pellet) were collected. The membrane fractions (pellet) were suspended in 20 μL of complete lysis buffer. The membrane fractions were quantified by BCA assay and analyzed by Western blot.

**Co-immunoprecipitation**

MH-0331-ME cell line (1 x 10^6^) was washed with PBS and lysed in immunoprecipitation buffer (150 mM NaCl, 100 mM Tris-HCl pH 8, 1% NP-40, protease, and phosphatase inhibitors) by gently pipetting. Protein A-magnetic beads (Thermo Fisher Scientific) were incubated 5 μg of rabbit anti-Rab11 IgG or 5 μg rabbit control IgG for 1 hour at 4 ºC in rotator. In all the conditions, the beads were washed three times with the washing buffer (150 mM NaCl, 100 mM Tris-HCl pH 8) on a magnetic rack, and then incubated overnight with 250 μg of whole cell lysate at 4 ºC on a rotator. The beads were washed three times with the immunoprecipitation buffer and then boiled in the sample buffer for 5 min at 95 ºC in a dry bath. All the samples collected were analyzed by Western blot. The antibodies and the dilutions utilized are described in **Table S3**.

**Indirect immunofluorescence**

MM cell lines (5 x 10^3^) were seeded in 8-well Falcon™ chambered culture slides (Thermo Fisher Scientific). The antibodies and the dilutions utilized are described in **Table S3**. Each slide was imaged using the Mantra Multispectral Imaging System (v1.0, Akoya Biosciences). The IFI30 and PD-L1 fluorescence intensities were estimated from 40X magnifying images using Qupath software (v.0.3.2, Queen’s University, Belfast, Northern Ireland). Fluorescence intensity was quantified automatically in the nuclear and cytosol areas of each cell after cell segmentation. The mean intensities in each condition were compared for statistical significance ^4^.

**Cycloheximide chase assay and chloroquine treatment**

MM cell lines (MH-0331-ME and SR-0788-ME) were co-incubated with 100 μg/mL of cycloheximide (#C7698; Millipore Sigma, Darmstadt, Germany) at different points (0, 20, 40, 60 min) and 200 UI/mL of IFN-γ (24 hours). In another assay, MM cell lines were co-incubated with 16 μM of chloroquine (Millipore Sigma, Darmstadt, Germany) at different points (0, 12, 24 hours) and 200 UI/mL of IFN-γ (24 hours). After respective incubation time, MM cell lines were harvested, processed for protein extraction, and Western blot analysis.

**Bioinformatics analysis**

The melanoma scRNA-seq dataset used as reference signature matrix for cell type deconvolution is publicly available (PMID34983745), and it was downloaded and used along with the code for CODEFACS provided by the authors ^5^. The reference signature matrices were applied to the TCGA-SKCM, PRJEB23709 ^6^, and PMID31792460 ^7^ public datasets. A total of 368 melanoma patients with RNA-Seq data for FFPE tissue samples from TCGA-SKCM dataset, 73 MM patients with RNA-Seq data for FFPE tissue samples from PRJEB23709 dataset, and 121 MM patients with RNA-Seq data for FFPE tissue samples from PMID31792460 dataset were imputed to estimate the cellular abundances and gene expression profiles in each cell type. Patients were stratified based on the median value of *IFI30* mRNA levels into high-*IFI30* and low-*IFI30* groups. OS, disease-specific survival (DSS), and PFS rates were determined by using Kaplan-Meier methods and significant differences were assessed using the Log-rank test using the R package survival (version 3.8-3) and survminer (version 0.5.1). MM patients from PMID31792460 and PRJEB23709 datasets were stratified based on response for ICI treatment into good or poor response groups and the levels of *IFI30* and *CD274* mRNA levels were compared between the two groups. PMID31792460 contains RNA-Seq data for 121 pre-treatment tumor tissue samples obtained from MM patients (stage III/IV) treated with ICIs (anti-PD-1). Patients were divided into good response (CR/SD/PR/MR with PFS > 1 year) and poor response (PD/SD/PR/MR with PFS ≤ 1 year) groups. PRJEB23709 dataset contains RNA-Seq data for 73 pre-treatment tumor tissue samples obtained from MM patients treated with ICIs (anti-PD-1 ± anti-CTLA4). Patients were stratified into good response (CR/PR/SD with PFS > 6 months) and poor response (PD/SD with PFS ≤ 6 months) groups. Differentially expressed gene (DEG) analysis was performed using DESeq2 (version 1.42.1) on raw feature counts.

Normal melanocyte data was obtained from GSE227015^8^. Briefly, STAR alignment^9^ was run with GRCh38v44, after the GSE227015 FASTQ files were trimmed using Trimmomatic v0.39. Featurecounts^10^ was used to perform gene read counts, and limma-voom differentially expressed genes (DEGs) analysis was conducted using the edgeR package (3.40.2). Limma-voom was selected in this case because we used the post-deconvolution malignant cell expression estimates for PRJEB23709 using a melanoma signature matrix adapted for CODEFACS usage, such that raw counts were unavailable. Data was quantile-normalized before comparison using the normalize.quantiles function available from preprocessCore (1.60.2).

**Supplementary Figures**


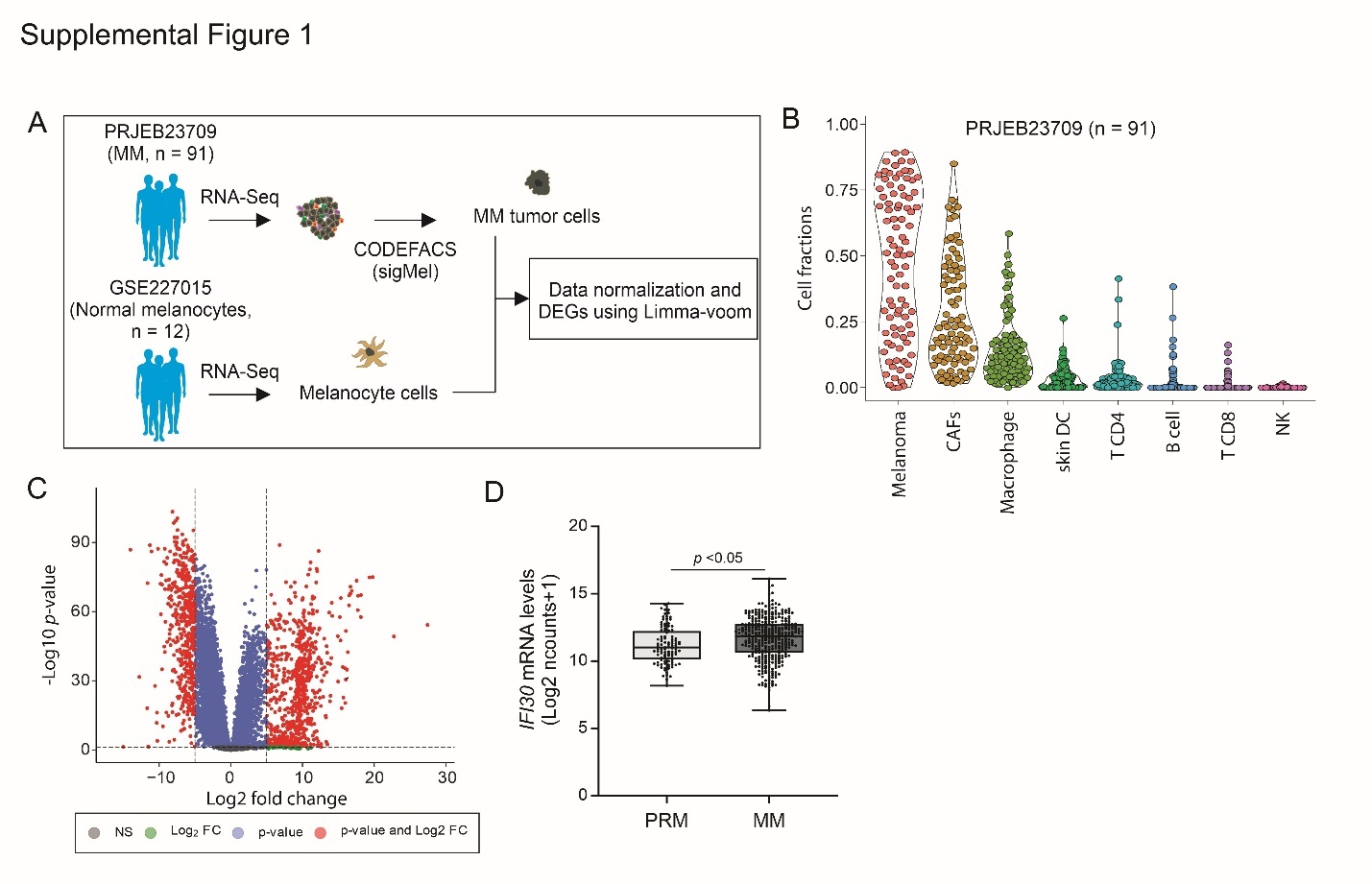


**Figure S1. TCGA-SKCM analysis in PRM and MM, and CODEFACs deconvolution and differential expression analysis in MM.** (A) Schematic representation of the datasets utilized and data normalization process comparing melanocytes cells (GSE227015 dataset) and metastatic melanoma (MM) cells (PRJEB23709 dataset after CODEFACs deconvolution analysis). (B) Cell fractions and cell types after CODEFACS deconvolution analysis of PRJEB23709 datasets. (C) Volcano plot shows the Log_10_ false discovery rate (FDR) of the p-values of the differentially expressed genes (DEGs) between normal melanocytes and MM cells that were described in S1B. (D) *IFI30* mRNA levels (log2 ncount+1) for primary melanoma (PRM) and metastatic melanoma (MM) from the TCGA-SKCM dataset. Statistical significance was assessed using t-test (D). Data represents the mean ± SD. NS, not significant.


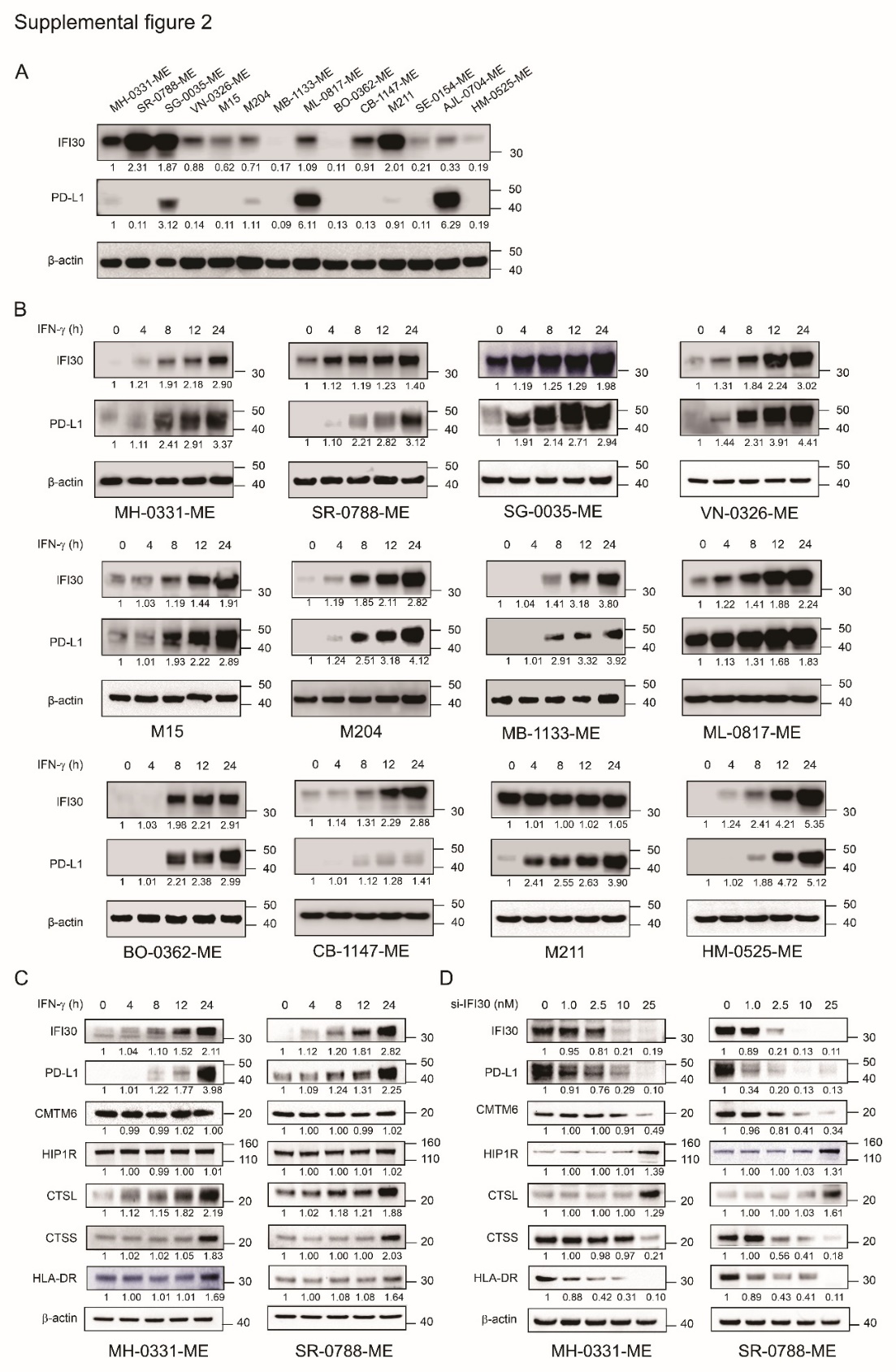


**Figure S2. IFI30 and PD-L1 protein levels are stimulated by IFN-γ in melanoma LNM cell lines.** (A) Western blot images show that IFI30 and PD-L1 expression without any treatment in melanoma lymph node metastasis derived cell lines (MH-0331-ME, SR-0788-ME, SG0035-ME, VN-0326-ME, M15, M204, MB-1133-ME, ML-0817-ME, BO-0362-Me, CB-1147-ME, M211, SE-0154-ME, AJL-0704-ME, and HM-0525-ME). (B) Western blot showing IFI30 and PD-L1 protein levels in MM cell lines (MH-0331-ME, SR-0788-ME, SG0035-ME, VN-0326-ME, M15, M204, MB-1133-ME, ML-0817-ME, BO-0362-Me, CB-1147-ME, M211, and HM-0525-ME) treated with 200 UI/mL IFN-γ for 24 hrs. (C) Western blot images show the changes in PD-L1, HIP1R, CMTM6, CSTL, CTSS protein levels over different times of IFN-γ treatment (0, 4, 8, 12, 24 hrs) in MM cell lines (MH-0031-ME and SR-0788-ME). (D) Western blot images show the changes in PD-L1, HIP1R, CMTM6, CSTL, CTSS protein levels using different doses of si-IFI30 in MM cell lines (MH-0031-ME and SR-0788-ME). β-actin was used as loading control in (A-D).


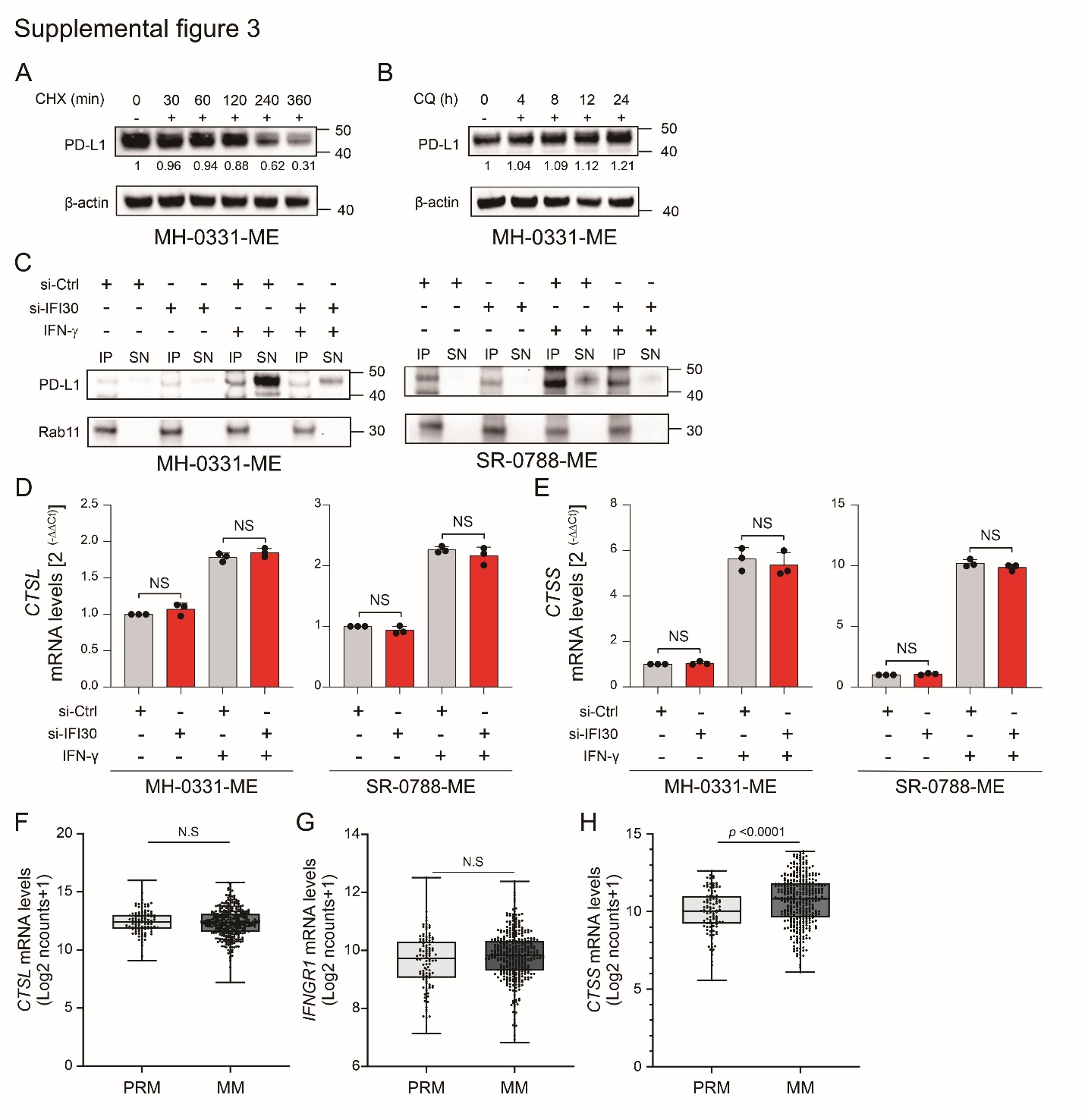


**Fig. S3. IFI30 regulates PD-L1 degradation in melanoma LNM cell lines.** (A-B) Western blot images show the changes in PD-L1 protein levels over different times (0, 30, 60, 120, 240, and 360 min) of exposure to cycloheximide (CHX, A) and chloroquine (0, 4, 8, 12, and 24 hours; CQ, B) treatment. (C) Immunoprecipitation assay using anti-Rab11 IgG and normal serum IgG. Westen blot images show PD-L1 levels in MM cell lines (MH-0031-ME and SR-0788-ME) of control (si-Ctrl) or *IFI30* knockdown (si-*IFI30*) untreated or treated with IFN-γ. β-actin was used as loading control in (A, B, C). (D-E) Bar plots show the mRNA levels of *CTSL* (D) and *CTSS* (E) in MM cell lines (MH-0031-ME and SR-0788-ME) of control (si-Ctrl) or *IFI30* knockdown (si-*IFI30*) untreated or treated with IFN-γ. (F-G) *CTSL* (F), *IFNGR1* (G), *CTSS* (H) mRNA levels (log2 ncount+1) for primary melanoma (PRM) and metastatic melanoma (MM) from the TCGA-SKCM dataset. Statistical significance was assessed using One-Way ANOVA and post-hoc test in (A, B) and t-test (D-H). Data represents the mean ± SD. NS, not significant.


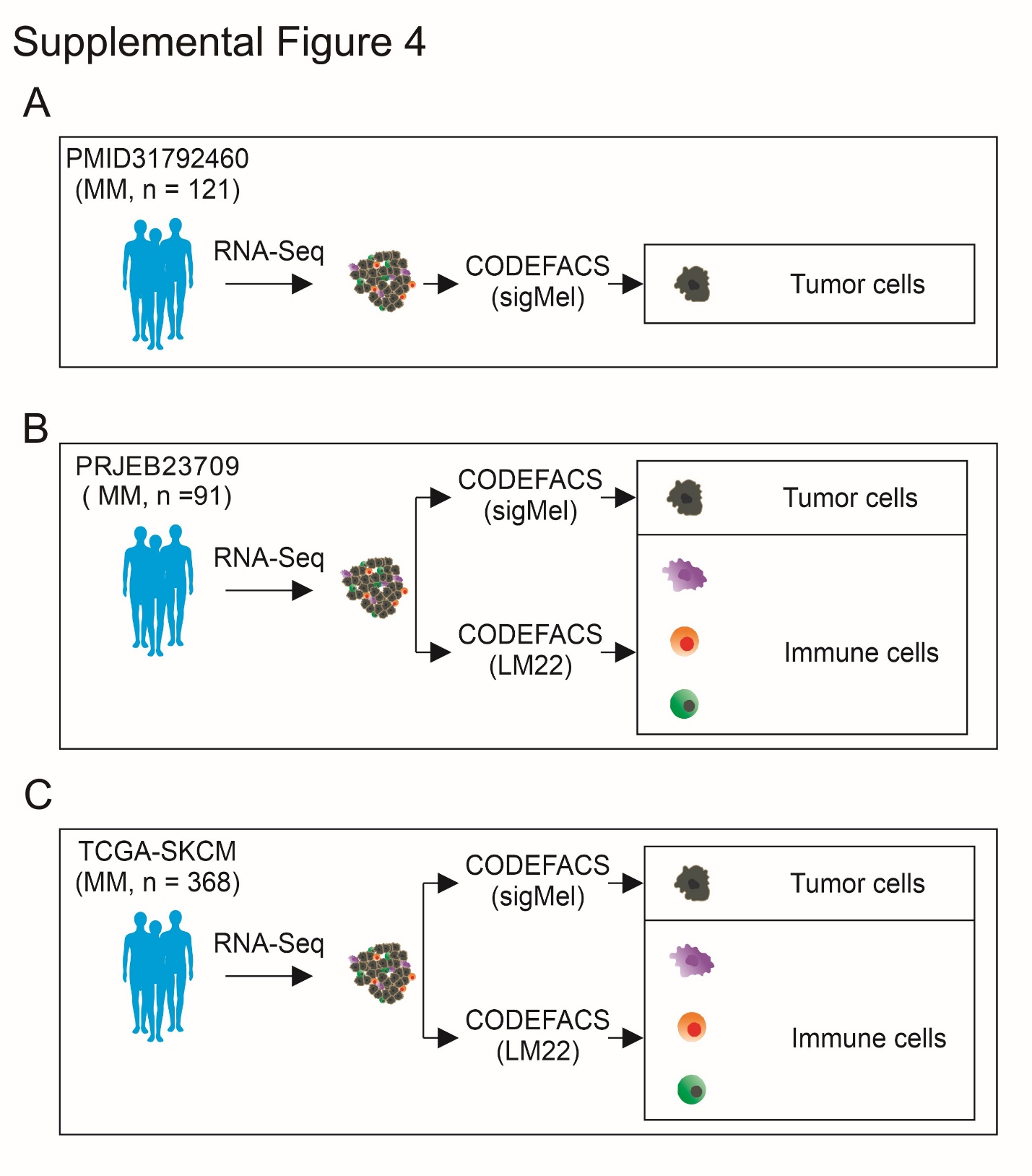


**Fig. S4. CODEFACs deconvolution in MM datasets.** (A-C) Schematic representations of the PMID31792460 (A), PRJEB23709 (B), and TCGA-SKCM (C) datasets analyzed by CODEFACs deconvolution analysis using sigMel or LM22 signature matrix.


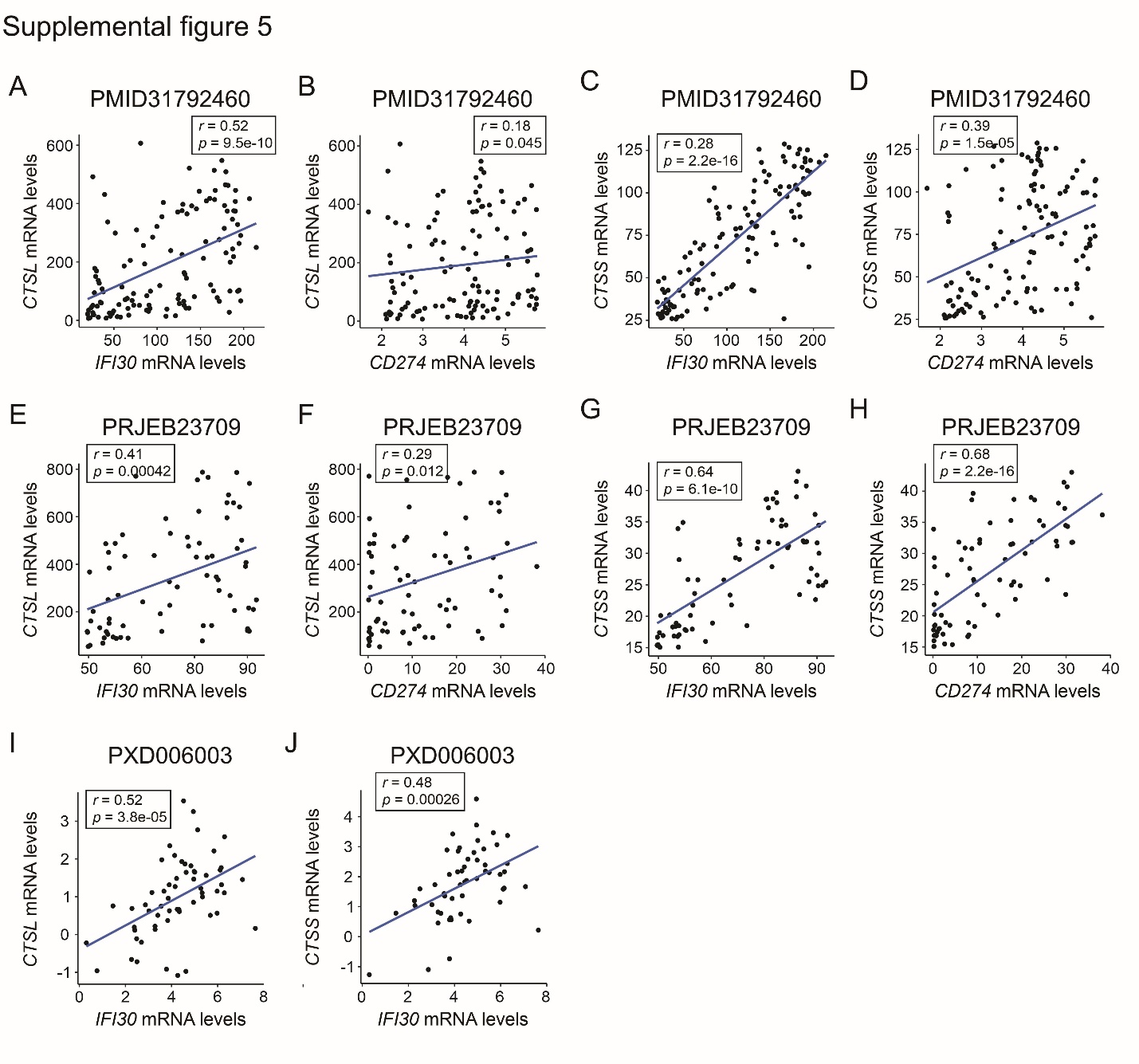


**Fig. S5. IFI30 correlates with CTSS and CTSL in metastatic melanoma.** (A-D) Scatter plots show the correlation between *IFI30* or *CD274* with *CTSL* (A, B) or *CTSS* (C, D) mRNA levels in melanoma cells after CODEFACS deconvolution analysis of PMID31792460 dataset. (E-H) Scatter plots show the correlation between *IFI30* or *CD274* with *CTSL* (E-F) or *CTSS* (G-H) mRNA levels in melanoma cells after CODEFACS deconvolution analysis of PRJEB23709 dataset. (I-J) Scatter plots show the correlation between IFI30 with CTSL (I) or CTSS (J) protein levels in tissue samples from PXD006003 dataset.


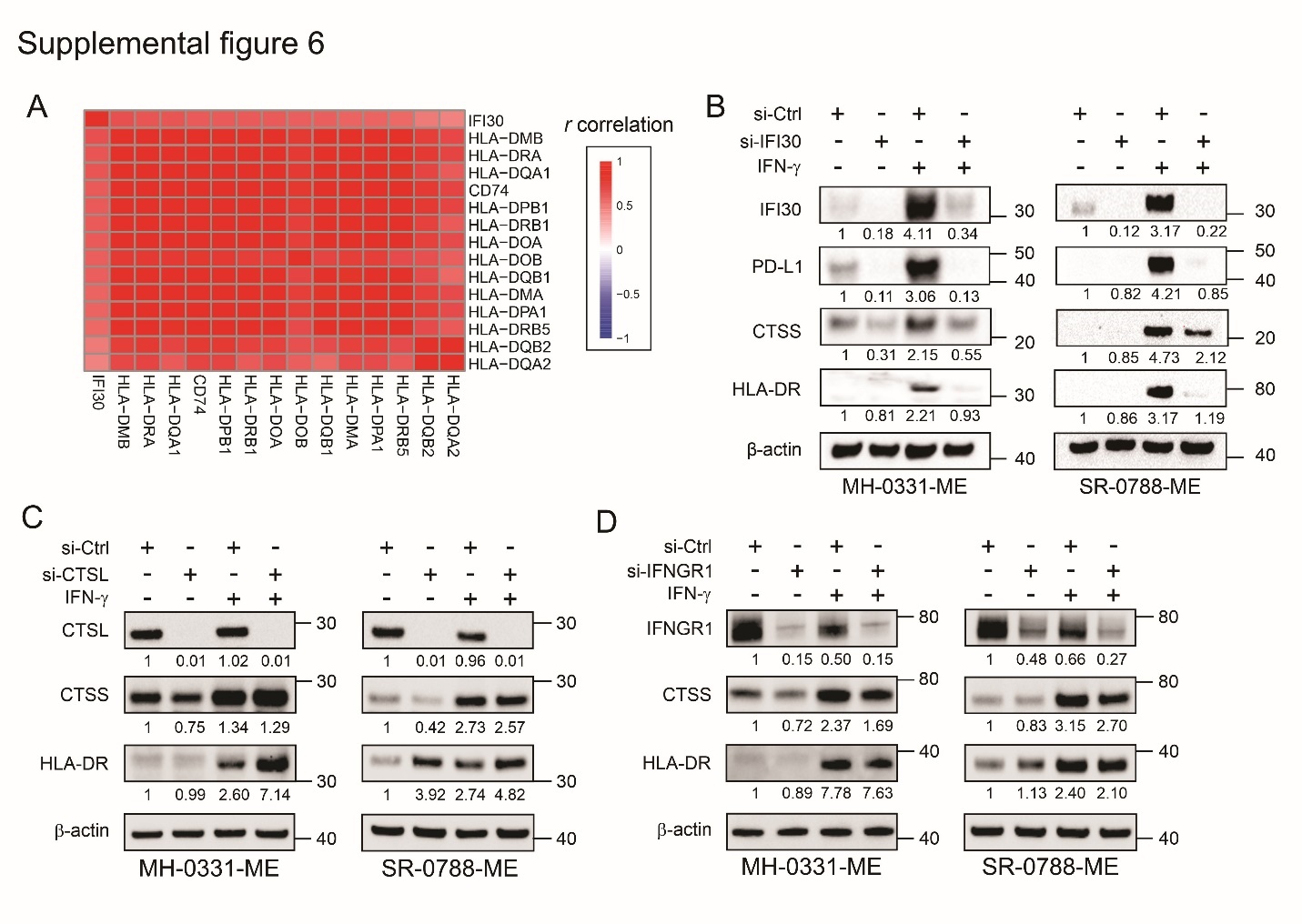


**Fig. S6. IFI30 and CTSL differentially regulate HLA-DR degradation in melanoma LNM cell lines.** (A) Heatmap shows the correlation between *IFI30* with MHC-II molecules in melanoma cells after CODEFACS deconvolution analysis of TCGA-SCKM MM (n =368) dataset. (B) Western blot images show the levels of IFI30, PD-L1, CTSS, and HLA-DR levels in MM cell lines (MH-0031-ME and SR-0788-ME) of control (si-Ctrl) or IFI30 knockdown (si-IFI30) untreated or treated with IFN-γ. (C) Western blot images show the levels of CTSL, CTSS, and HLA-DR levels in MM cell lines (MH-0031-ME and SR-0788-ME) of control (si-Ctrl) or CTSL knockdown (si-CTSL) untreated or treated with IFN-γ. (D) Western blot images show the levels of IFNGR1, CTSS, and HLA-DR levels in MM cell lines (MH-0031-ME and SR-0788-ME) of control (si-Ctrl) or IFNGR1 knockdown (si-IFNGR1) untreated or treated with IFN-γ.

**
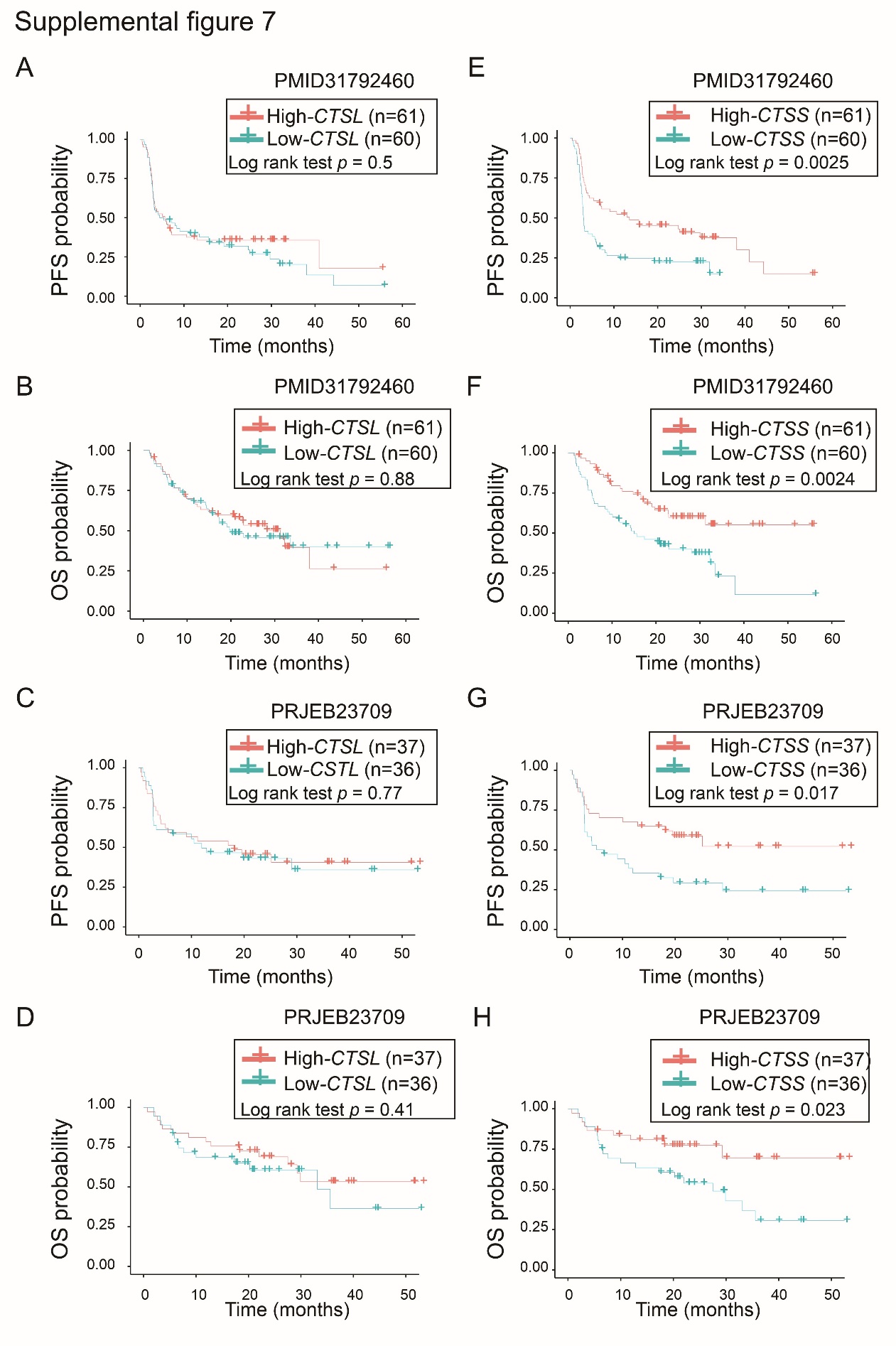
**

**Fig. S7. CTSS but not CTSL is associated with clinical outcomes in MM patients.** (A-B) Kaplan-Meier curves show the progression-free survival (PFS, A) and overall survival (OS, B) probability comparing MM patients with high- vs low-CTSL from the PMID31792460 dataset. (C-D) Kaplan-Meier curves show the PFS (C) and OS (B) probability comparing MM patients with high- vs low-CTSL from the PRJEB23709 dataset. (E-F) Kaplan-Meier curves show the PFS (E) and OS (F) probability comparing MM patients with high- vs low-CTSS from the PMID31792460 dataset. (G-H) Kaplan-Meier curves show the PFS (G) and OS (H) probability comparing MM patients with high- vs low-CTSS from the PRJEB23709 dataset. Log-rank test in (A-H).

**References**

1. Furuhashi S, Bustos MA, Mizuno S, et al. Spatial profiling of cancer-associated fibroblasts of sporadic early onset colon cancer microenvironment. NPJ Precis Oncol. 2023;7(1):118. doi: 10.1038/s41698-023-00474-w.

2. Murakami T, Shoji Y, Nishi T, et al. Regulation of MRE11A by UBQLN4 leads to cisplatin resistance in patients with esophageal squamous cell carcinoma. Mol Oncol. 2021;15(4):1069-1087. doi: 10.1002/1878-0261.12929.

3. Kobayashi Y, Bustos MA, Hayashi Y, et al. Interferon-induced factor 16 is essential in metastatic melanoma to maintain STING levels and the immune responses upon IFN-γ response pathway activation. J Immunother Cancer. 2024;12(10). doi: 10.1136/jitc-2024-009590.

4. Mizuno S, Bustos MA, Hayashi Y, et al. Induced collagen type-I secretion by hepatocytes of the melanoma liver metastasis is associated with a reduction in tumour-infiltrating lymphocytes. Clin Transl Med. 2024;14(11):e70067. doi: 10.1002/ctm2.70067.

5. Wang K, Patkar S, Lee JS, et al. Deconvolving Clinically Relevant Cellular Immune Cross-talk from Bulk Gene Expression Using CODEFACS and LIRICS Stratifies Patients with Melanoma to Anti-PD-1 Therapy. Cancer Discov. 2022;12(4):1088-1105. doi: 10.1158/2159-8290.Cd-21-0887.

6. Gide TN, Quek C, Menzies AM, et al. Distinct Immune Cell Populations Define Response to Anti-PD-1 Monotherapy and Anti-PD-1/Anti-CTLA-4 Combined Therapy. Cancer Cell. 2019;35(2):238-255.e6. doi: 10.1016/j.ccell.2019.01.003.

7. Liu D, Schilling B, Liu D, et al. Integrative molecular and clinical modeling of clinical outcomes to PD1 blockade in patients with metastatic melanoma. Nature medicine. 2019;25(12):1916-1927. doi: 10.1038/s41591-019-0654-5.

8. Bajpai VK, Swigut T, Mohammed J, et al. A genome-wide genetic screen uncovers determinants of human pigmentation. Science. 2023;381(6658):eade6289. doi: 10.1126/science.ade6289.

9. Dobin A, Davis CA, Schlesinger F, et al. STAR: ultrafast universal RNA-seq aligner. Bioinformatics. 2013;29(1):15-21. doi: 10.1093/bioinformatics/bts635.

10. Liao Y, Smyth GK, Shi W. featureCounts: an efficient general purpose program for assigning sequence reads to genomic features. Bioinformatics. 2014;30(7):923-30. doi: 10.1093/bioinformatics/btt656.
