# Supplementary material for: Interferon-gamma-inducible protein 30 prevents IFN-γ-receptor 1 degradation to maintain PD-L1 and MHC-II levels in metastatic melanoma
Source: Cell Commun Signal. 2026 Feb 12;24:182. doi: 10.1186/s12964-026-02710-9 (PMC12998310; doi:10.1186/s12964-026-02710-9)
Supplement: Supplementary file 3 — Supplementary Material 3. [file 12964_2026_2710_MOESM3_ESM.docx]

**Interferon-gamma-inducible protein 30 prevents IFN-γ-receptor 1 degradation to maintain PD-L1 and MHC-II levels in metastatic melanoma**

Shodai Mizuno^1#^, Yuka Mizuno^1#^, Kodai Abe^1^, Anne M. Macy^2,3^, Kelly Chong^1^, Yuta Kobayashi^1^, Karen T. Hastings^2,3,4^, Dave S. B. Hoon^1, 3^ and Matias A. Bustos^1^

**Affiliations of authors:**

^1^Department of Translational Molecular Medicine, Saint John’s Cancer Institute (SJCI), Providence Saint John’s Health Center (SJHC), Santa Monica, CA 90404, USA.

^2^Phoenix Veterans Affairs Health Care System, Phoenix, AZ, USA.

^3^Department of Dermatology, College of Medicine - Phoenix, University of Arizona, Phoenix, AZ, USA.

^4^University of Arizona Cancer Center, University of Arizona, Tucson, AZ, USA.

^5^Department of Genome Sequencing Center, SJCI, Providence SJHC, Santa Monica, CA 90404, USA.

# These authors contributed equally.

**Address correspondence and reprint requests to:**

Matias A. Bustos; Dept. of Translational Molecular Medicine, SJCI, Providence SJHC, 2200 Santa Monica Blvd, Santa Monica, CA, 90404, USA.

Email: matias.bustos@providence.org

**Conflict of Interest**

The authors declare no conflicts of interest.

**
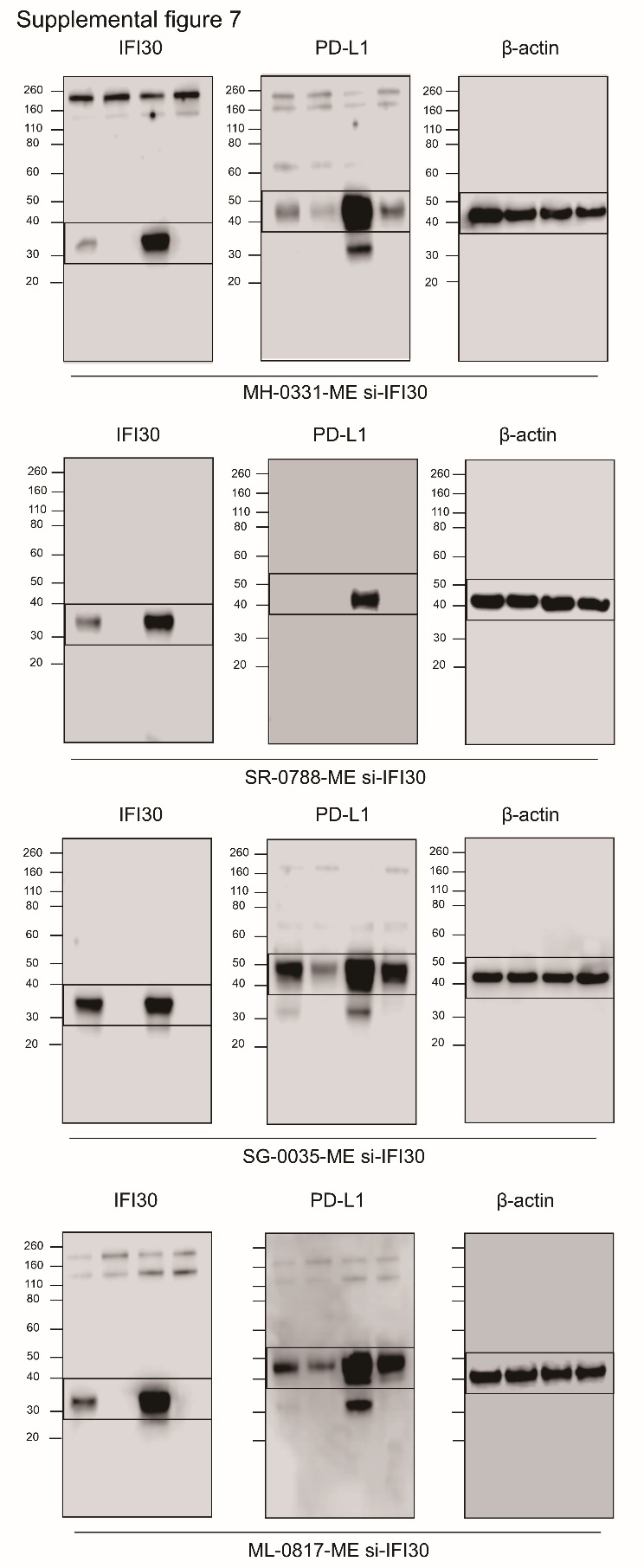
**

**Uncropped western blot images.** Uncropped western blot images for Figure 1O.


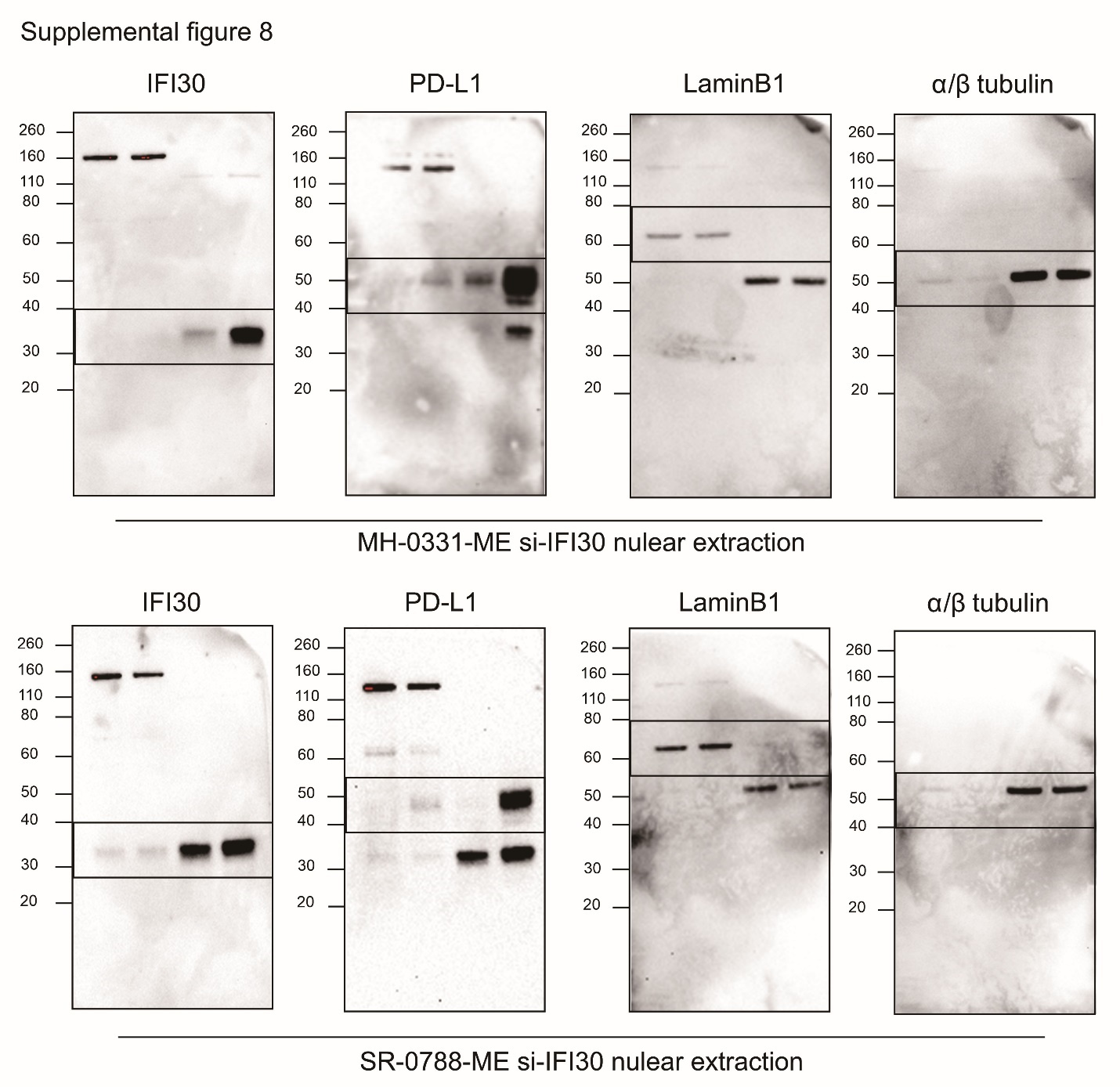


**Uncropped western blot images.** Uncropped western blot images for Figure 1R.

**
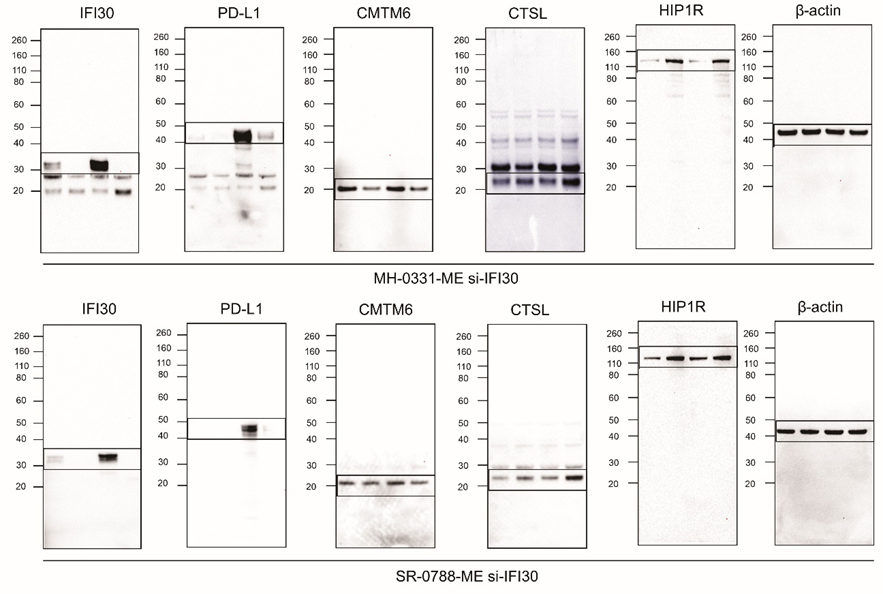
**

**Uncropped western blot images.** Uncropped western blot images for Figure 2A.

**
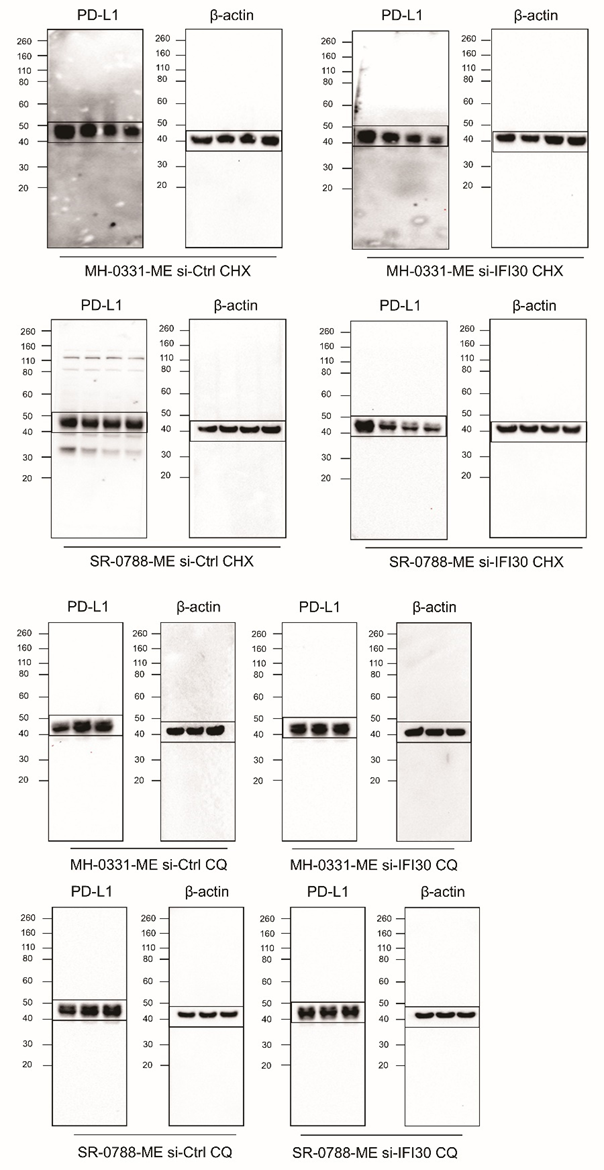
**

**Uncropped western blot images.** Uncropped western blot images for Figure 2B.

**
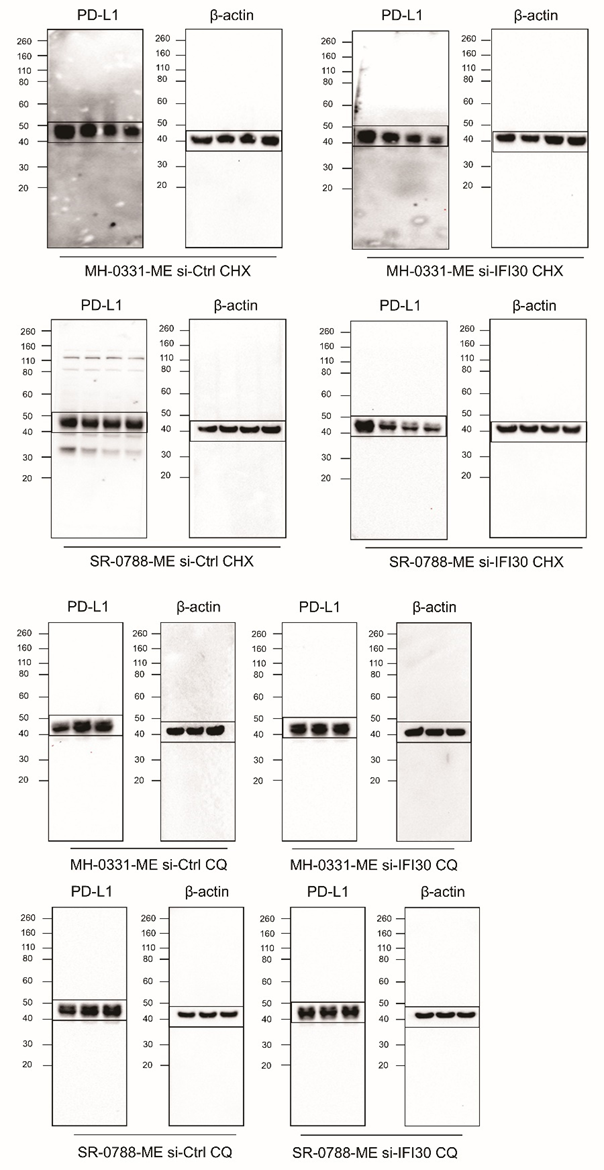
**

**Uncropped western blot images.** Uncropped western blot images for Figure 2C.

**
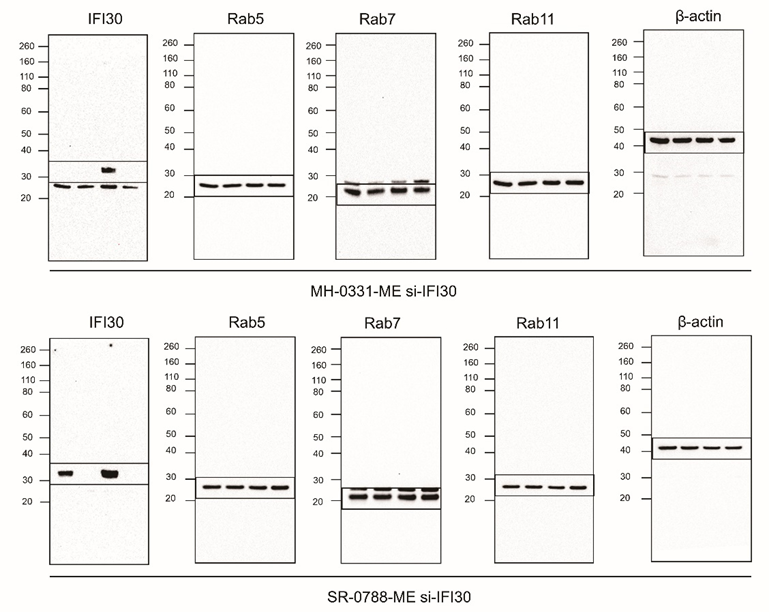
**

**Uncropped western blot images.** Uncropped western blot images for Figure 2D.

**
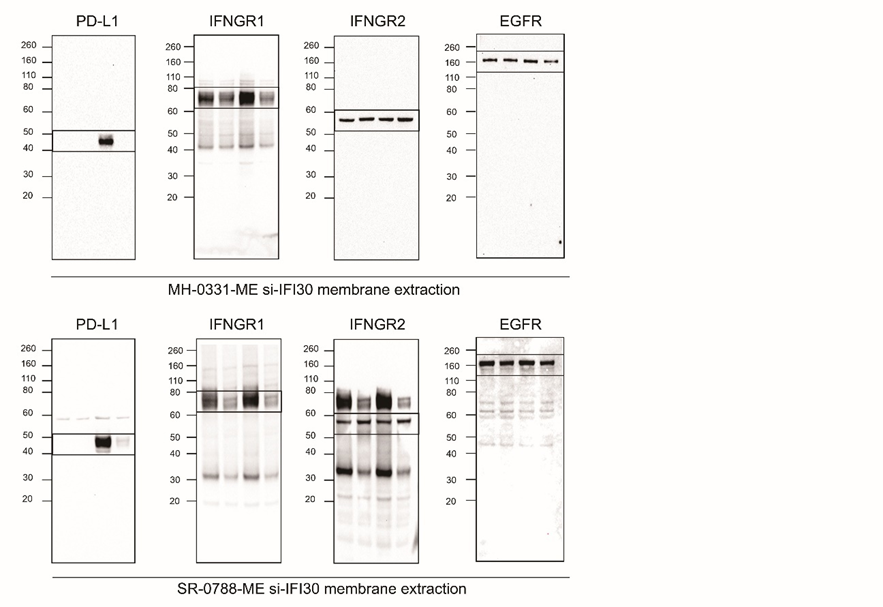
**

**Uncropped western blot images.** Uncropped western blot images for Figure 2E.

**
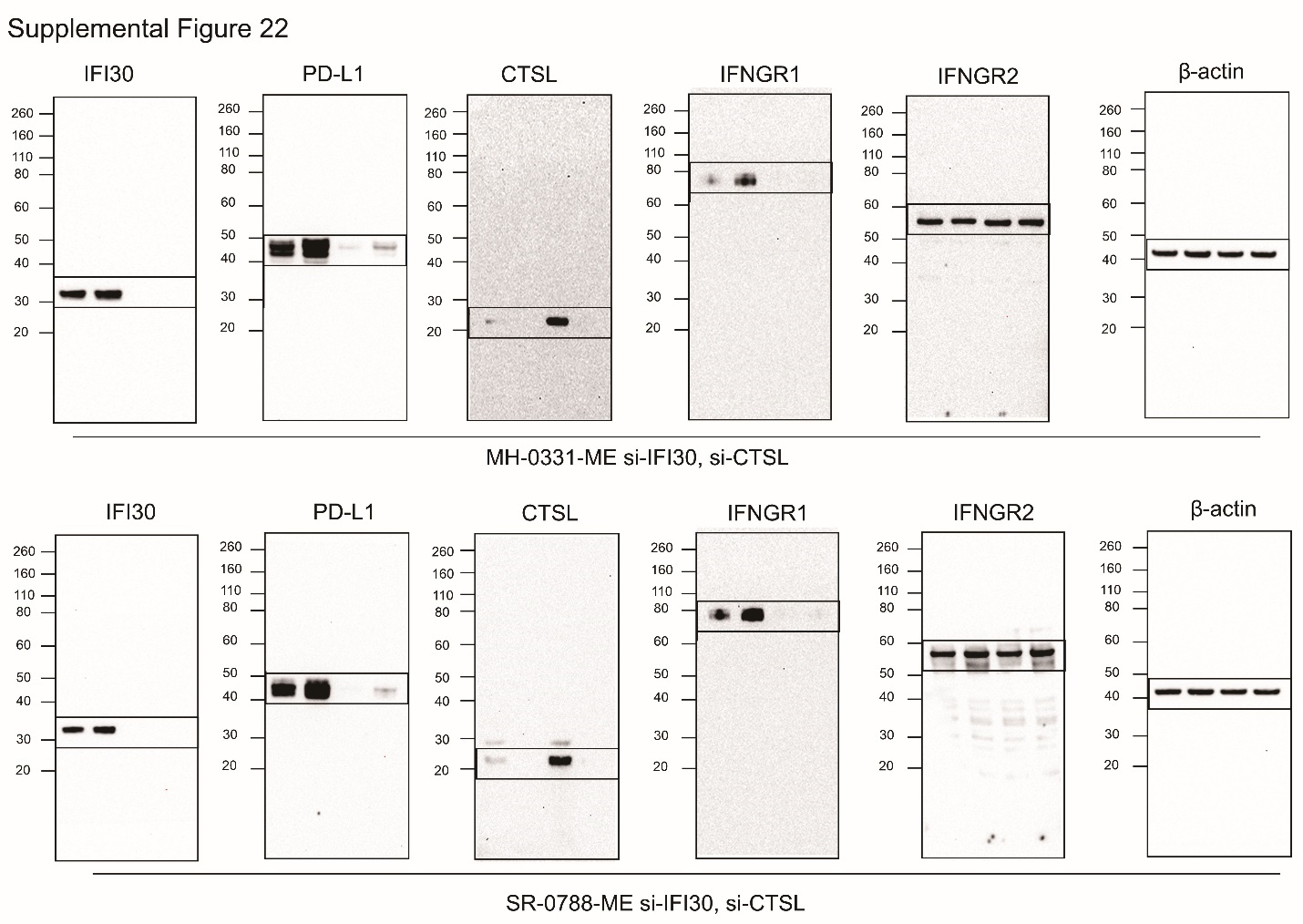
**

**Uncropped western blot images.** Uncropped western blot images for Figure 2F.


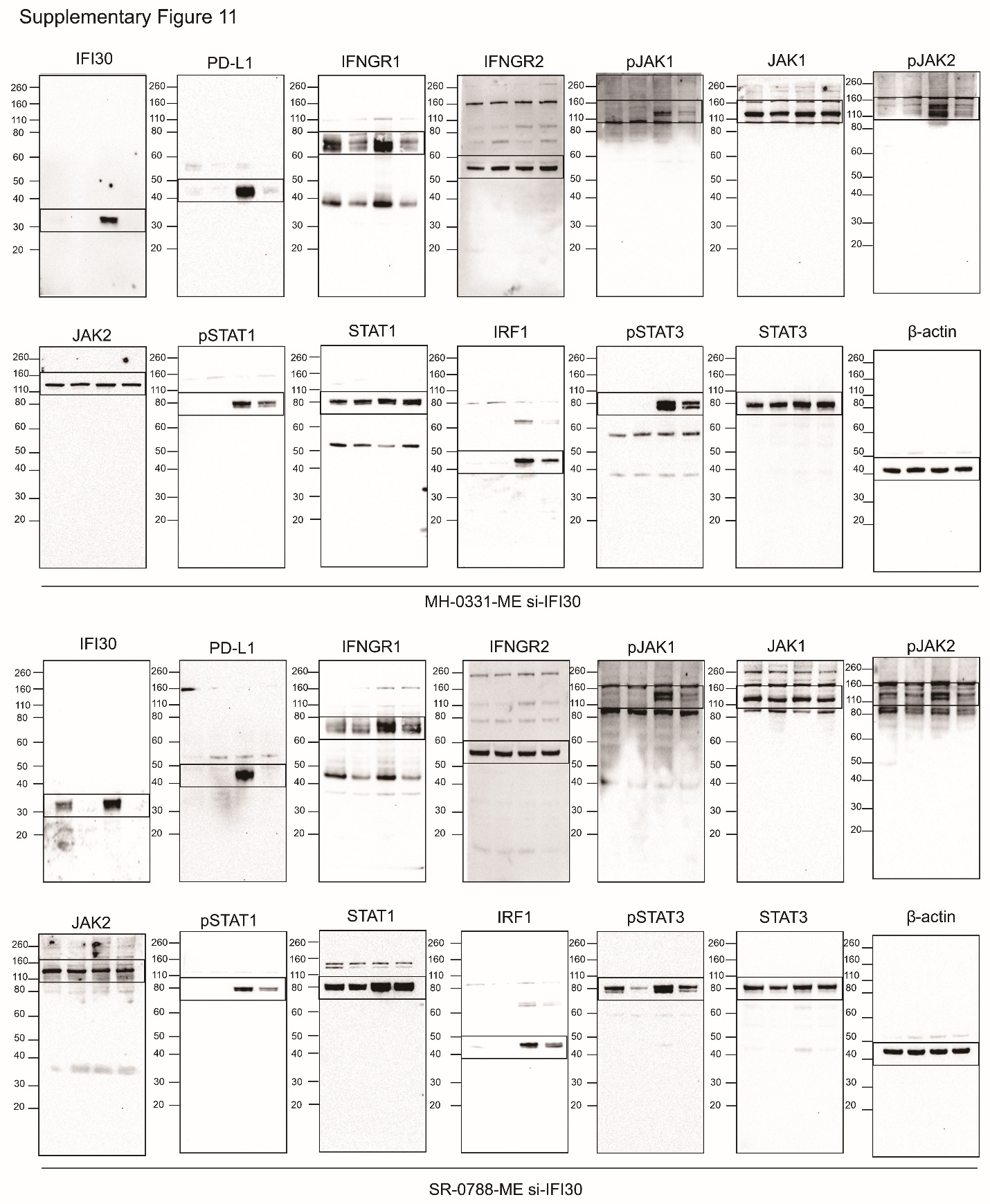
**
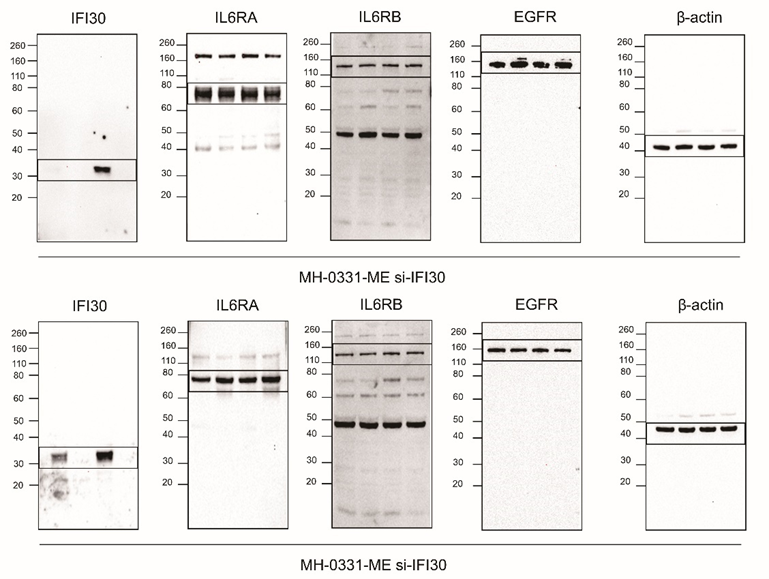
**

**Uncropped western blot images.** Uncropped western blot images for Figure 3C.

**
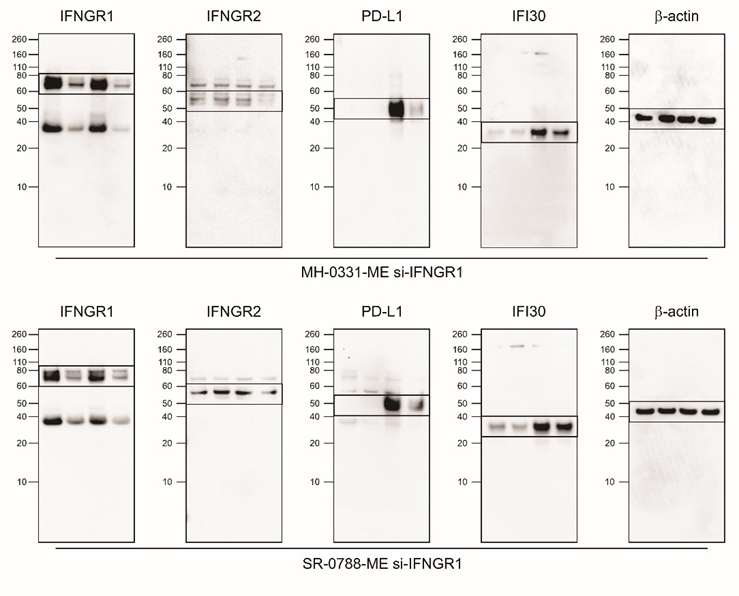
**

**Uncropped western blot images.** Uncropped western blot images for Figure 4A.


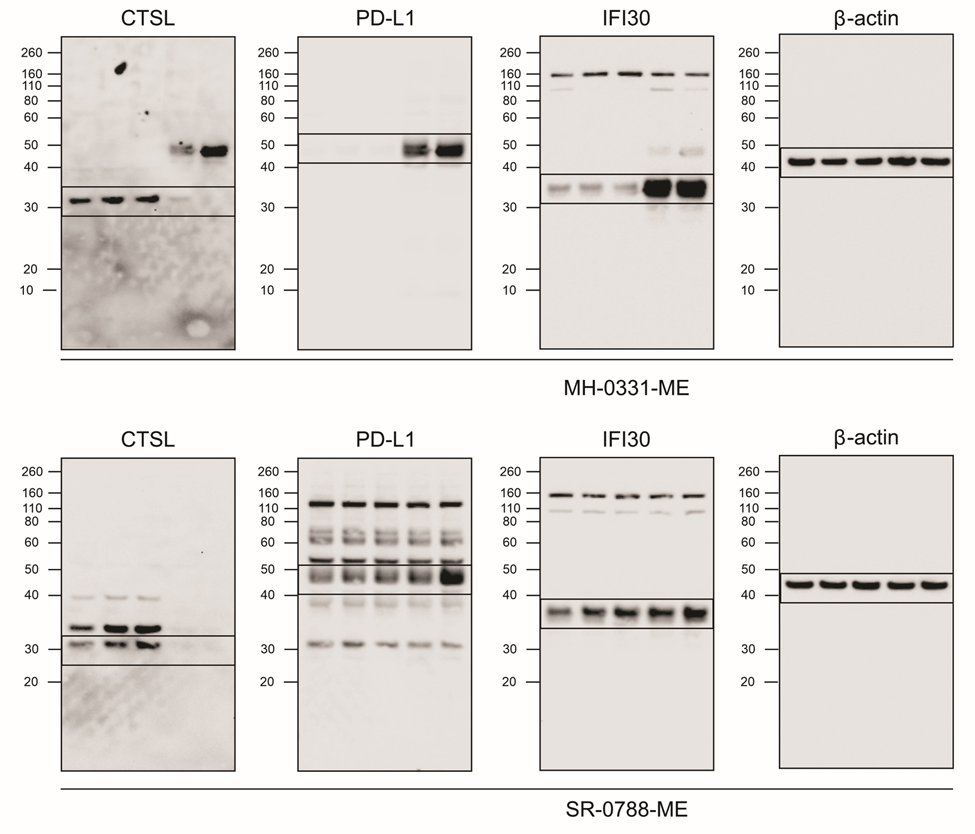


**Uncropped western blot images.** Uncropped western blot images for Figure 4D.


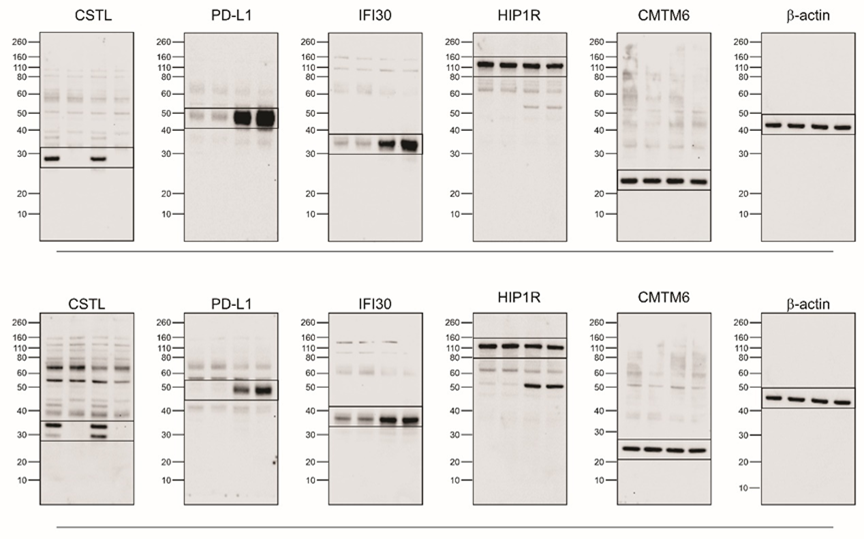


**Uncropped western blot images.** Uncropped western blot images for Figure 4E.

**
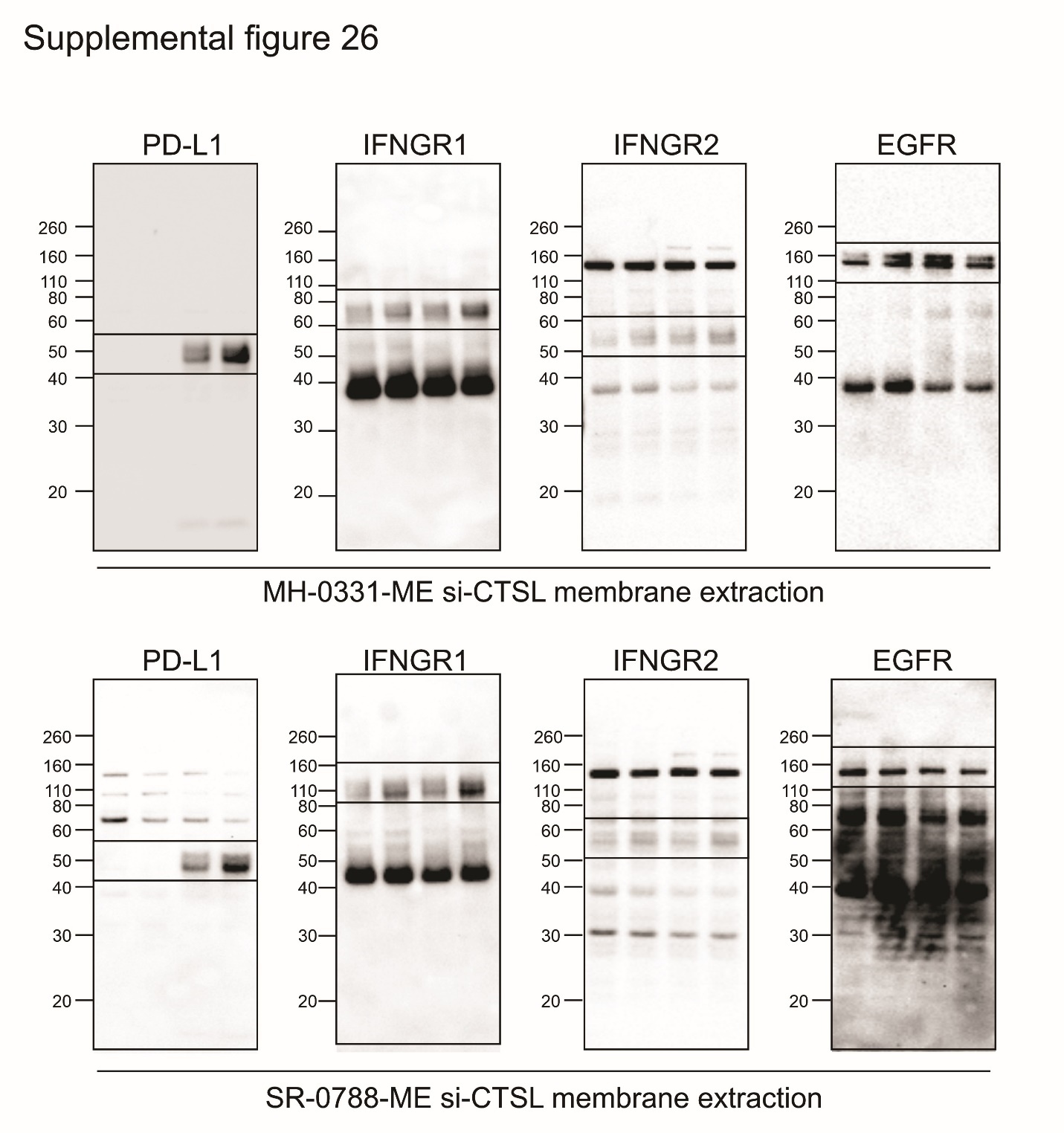
Uncropped western blot images.** Uncropped western blot images for Figure 4F.

**
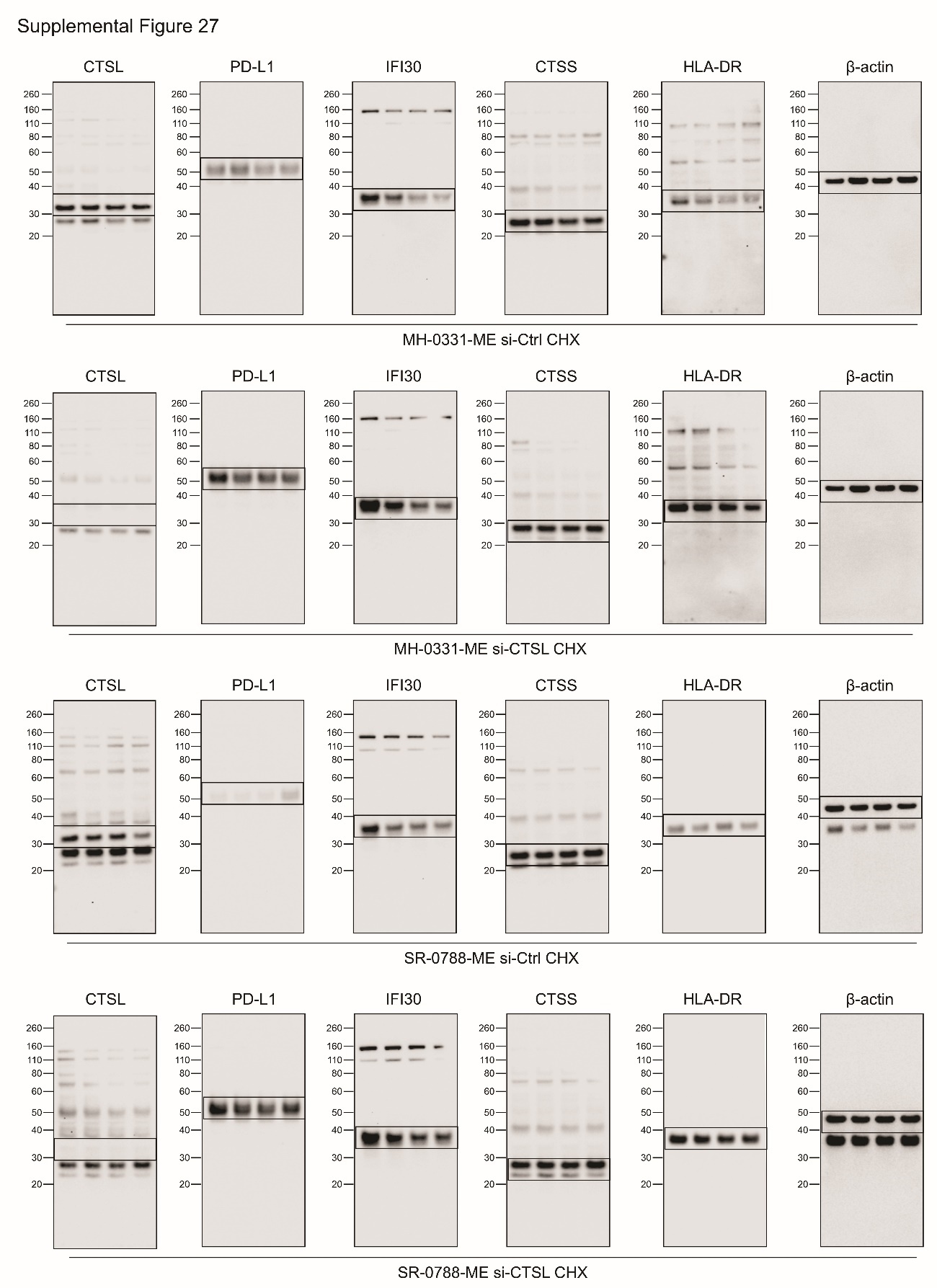
**

**Uncropped western blot images.** Uncropped western blot images for Figure 4G.

**
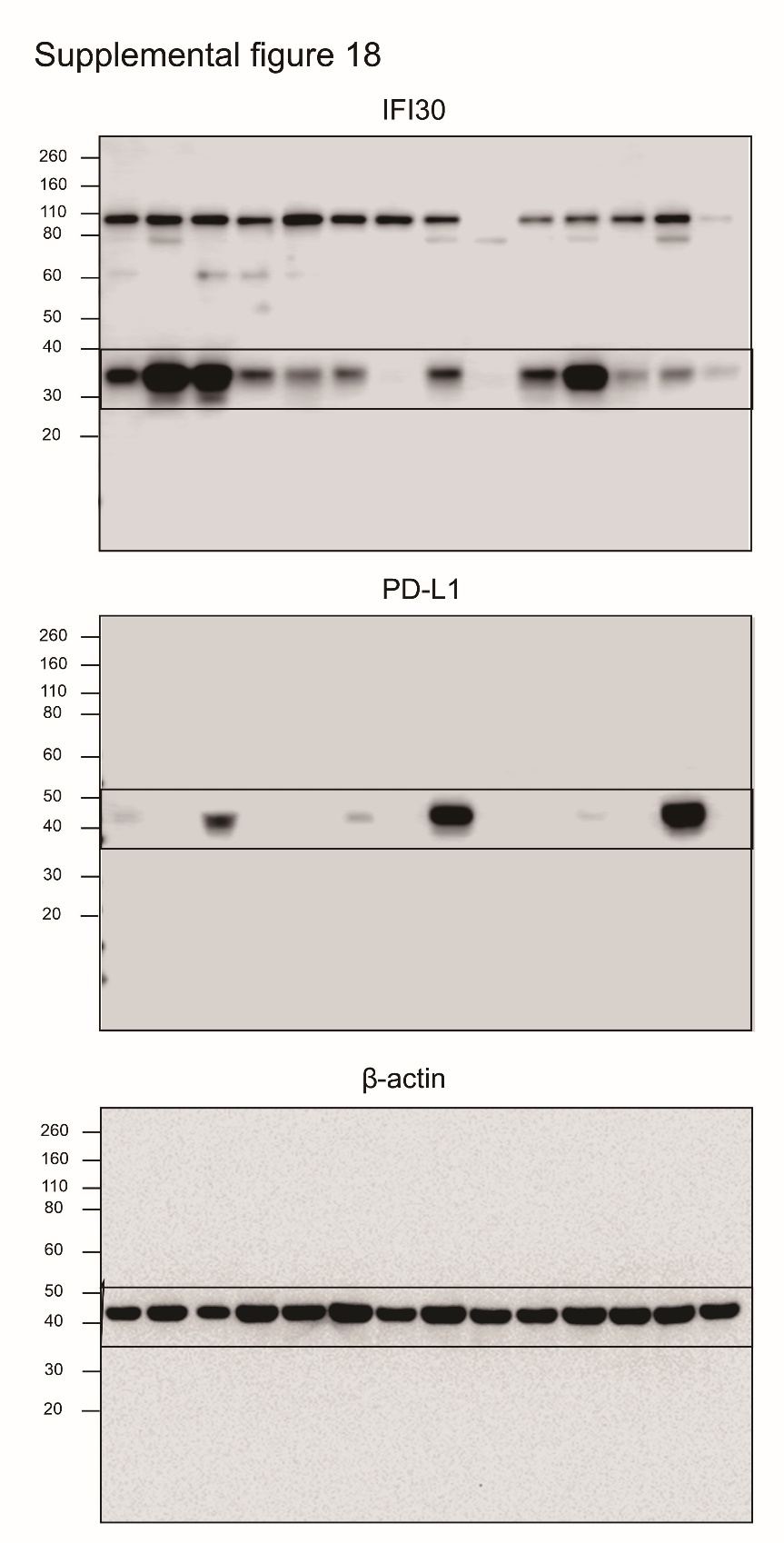
**

**Uncropped western blot images.** Uncropped western blot images for Figure S2A


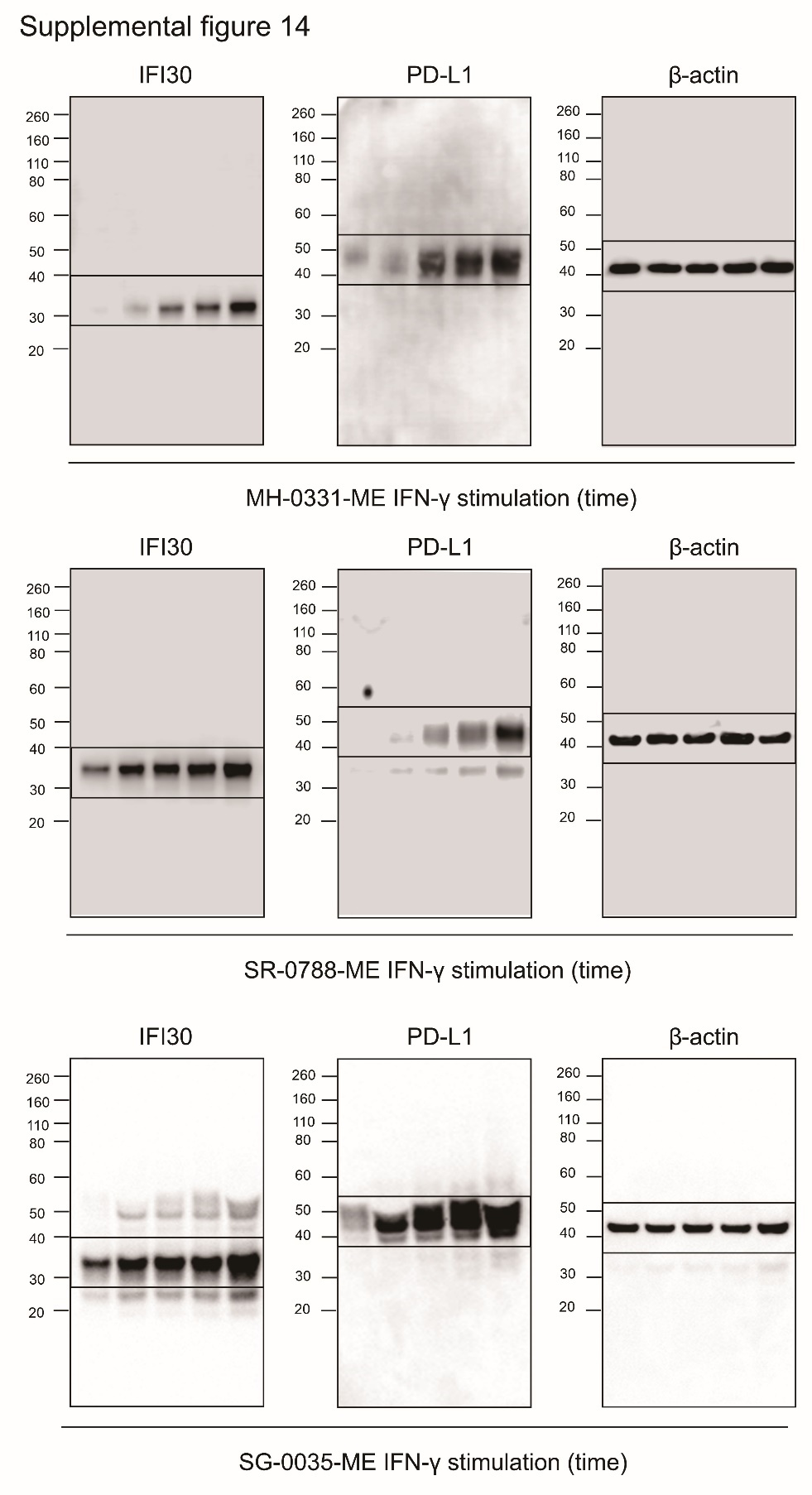


**Uncropped western blot images.** Uncropped western blot images for Figure S2B.


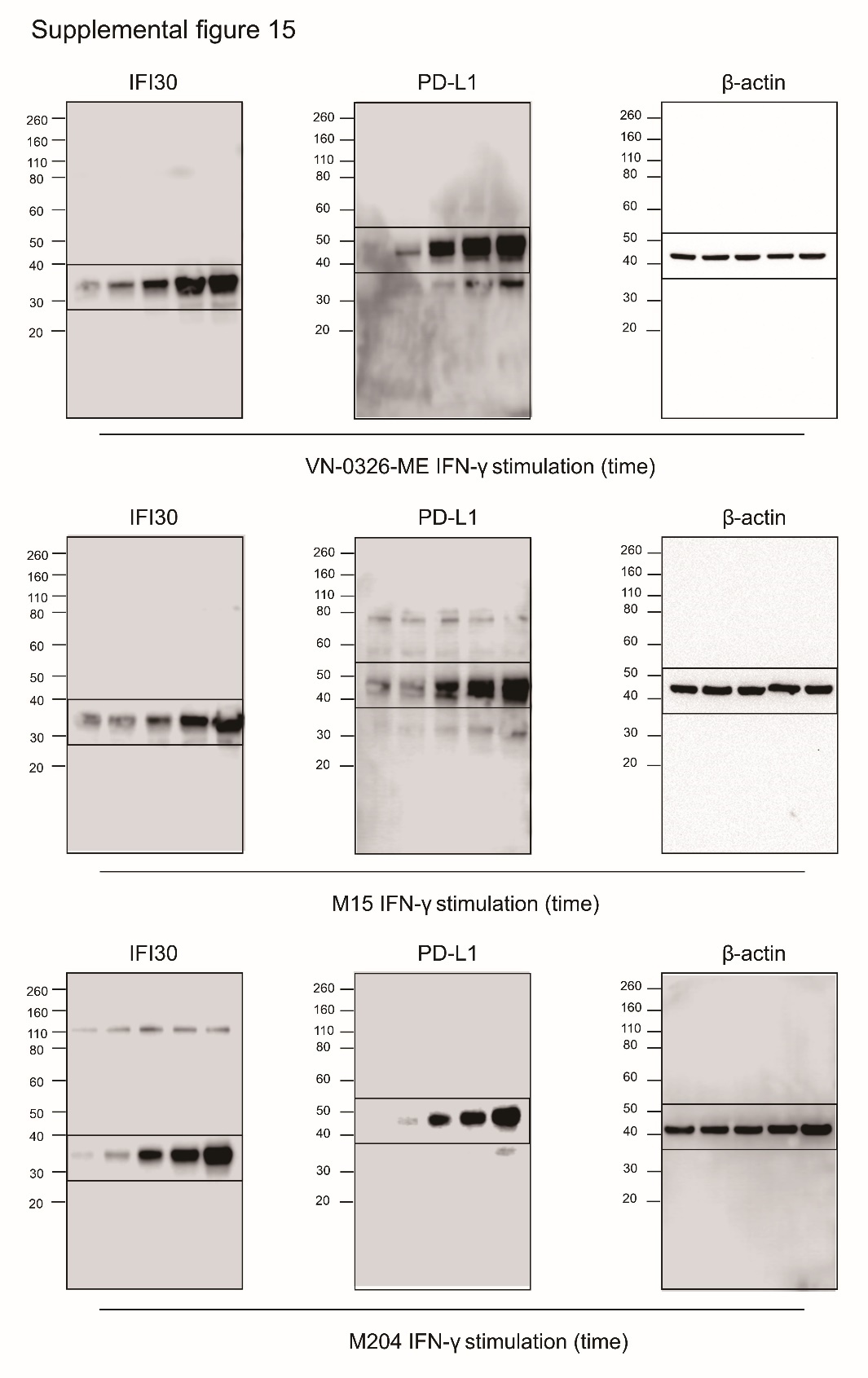


**Uncropped western blot images.** Uncropped western blot images for Figure S2B.

**
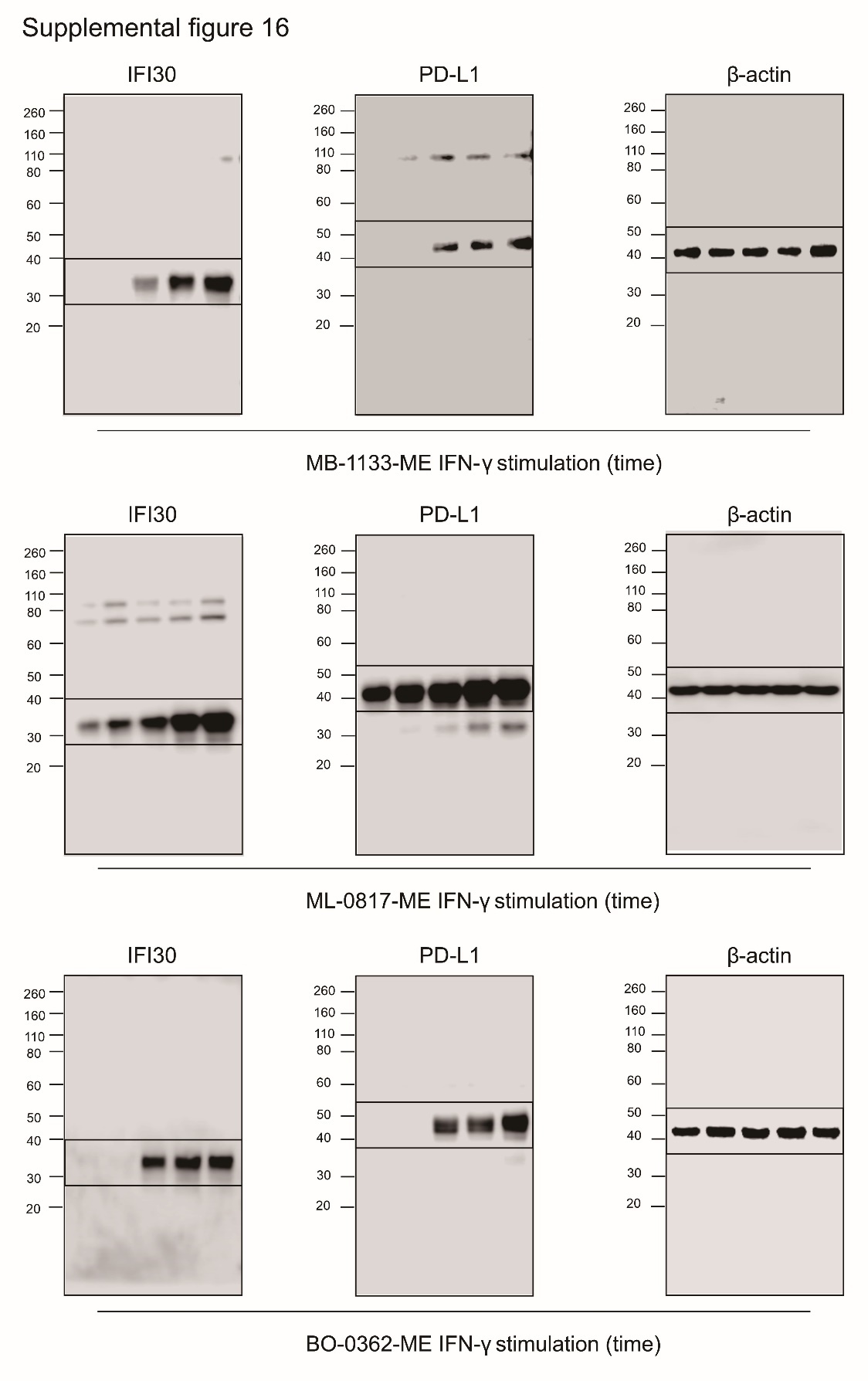
**

**Uncropped western blot images.** Uncropped western blot images for Figure S2B.

**
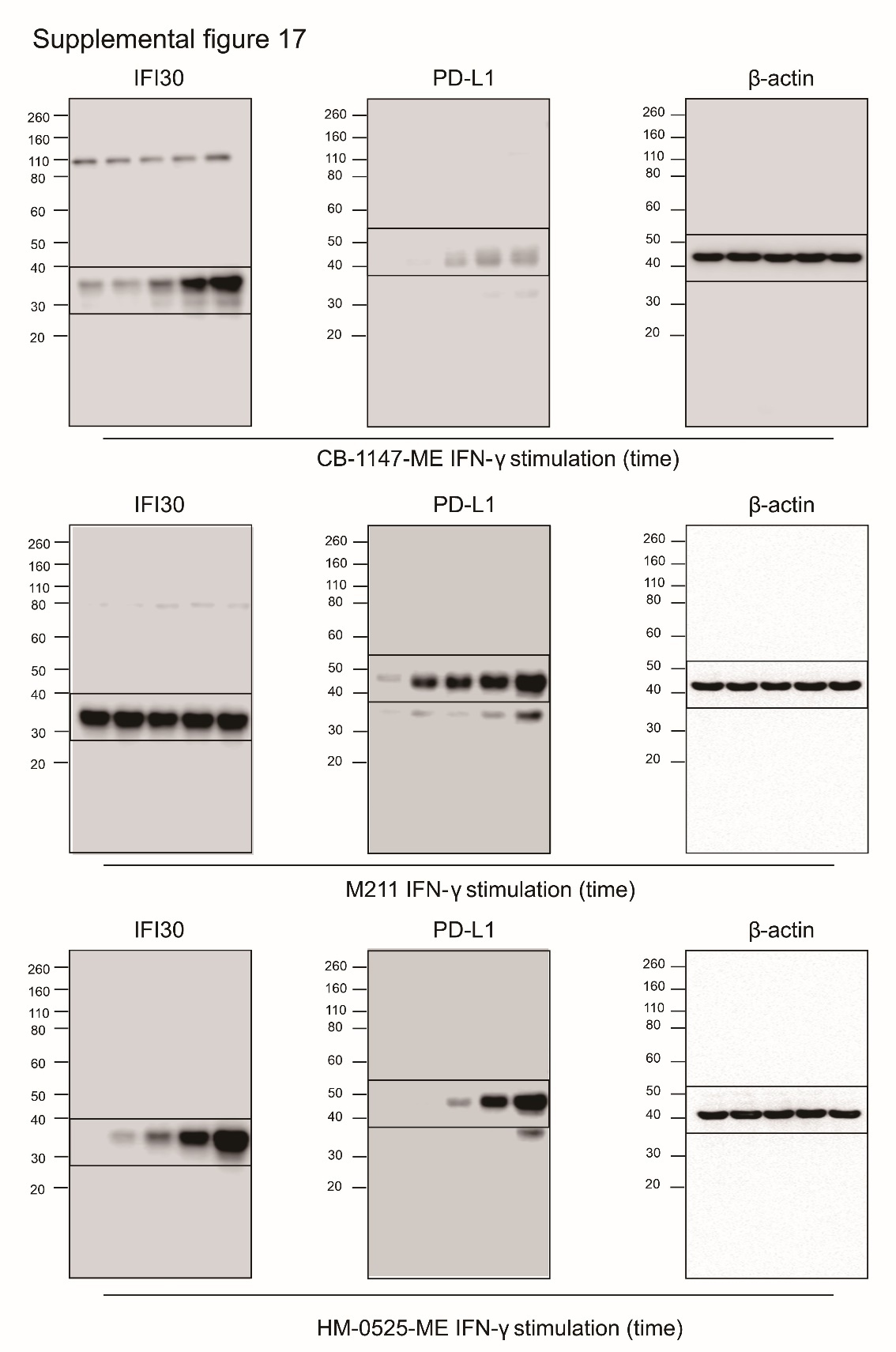
**

**Uncropped western blot images.** Uncropped western blot images for Figure S2B.


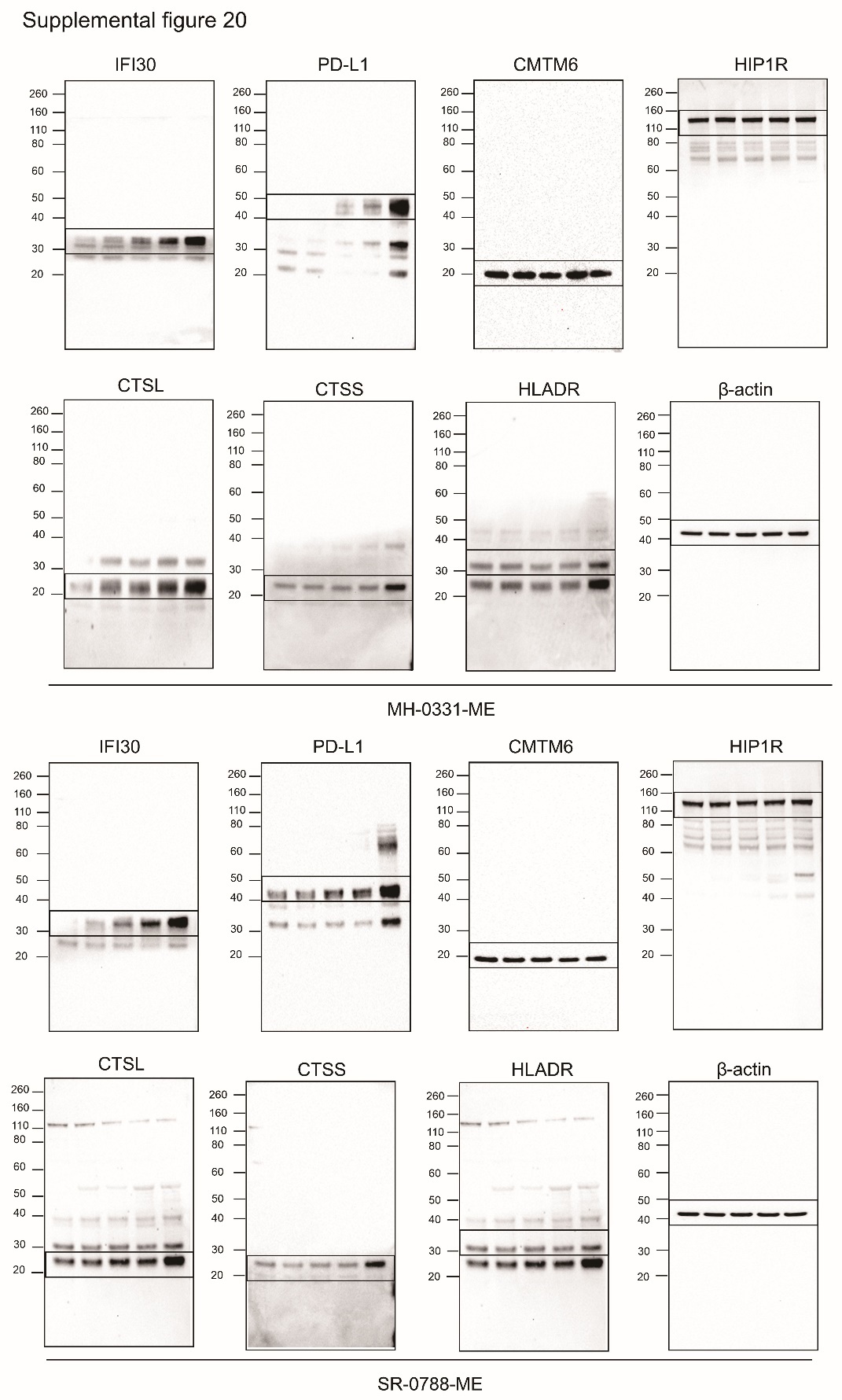


**Uncropped western blot images.** Uncropped western blot images for Figure S2C.

**
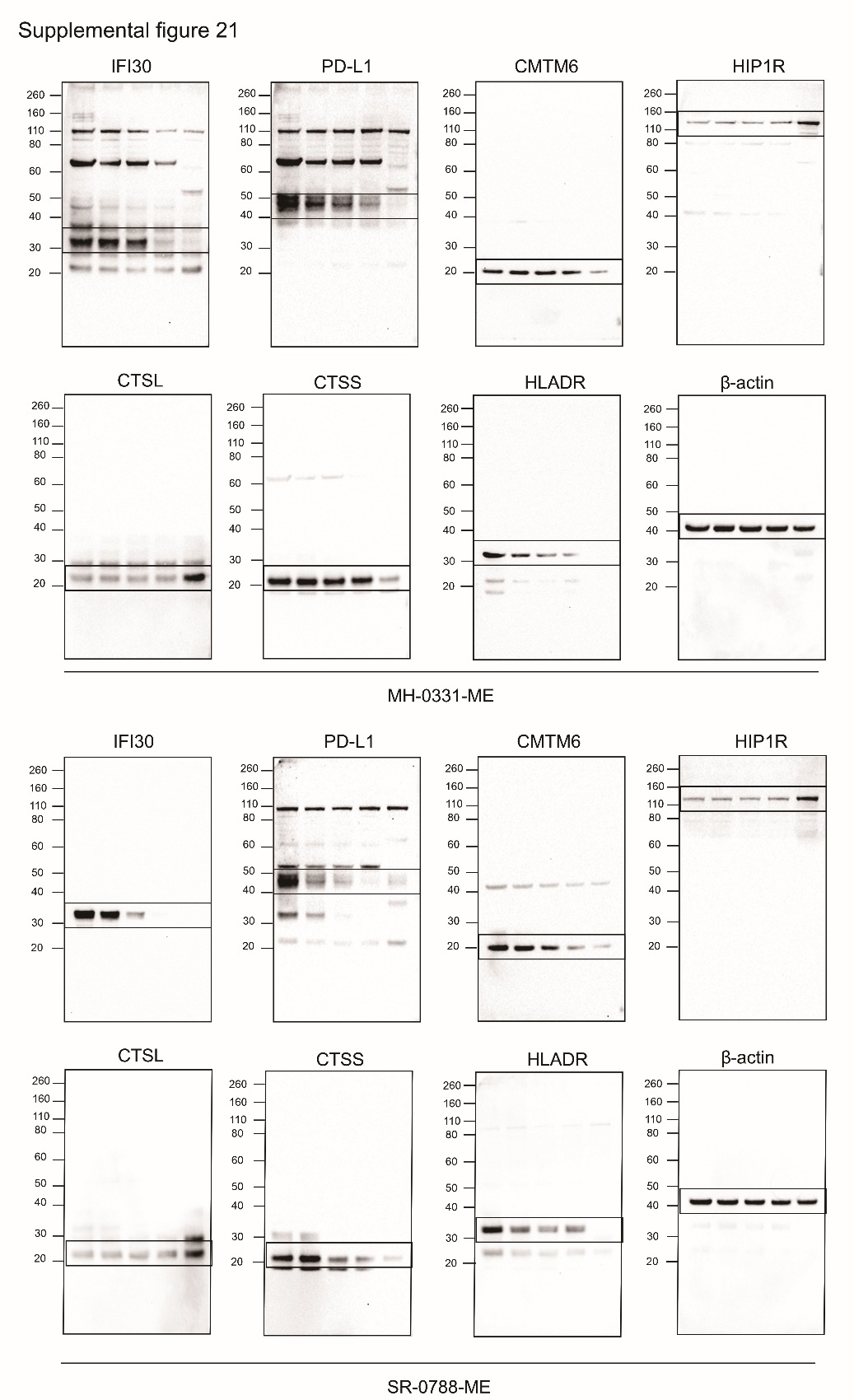
**

**Uncropped western blot images.** Uncropped western blot images for Figure S2D.


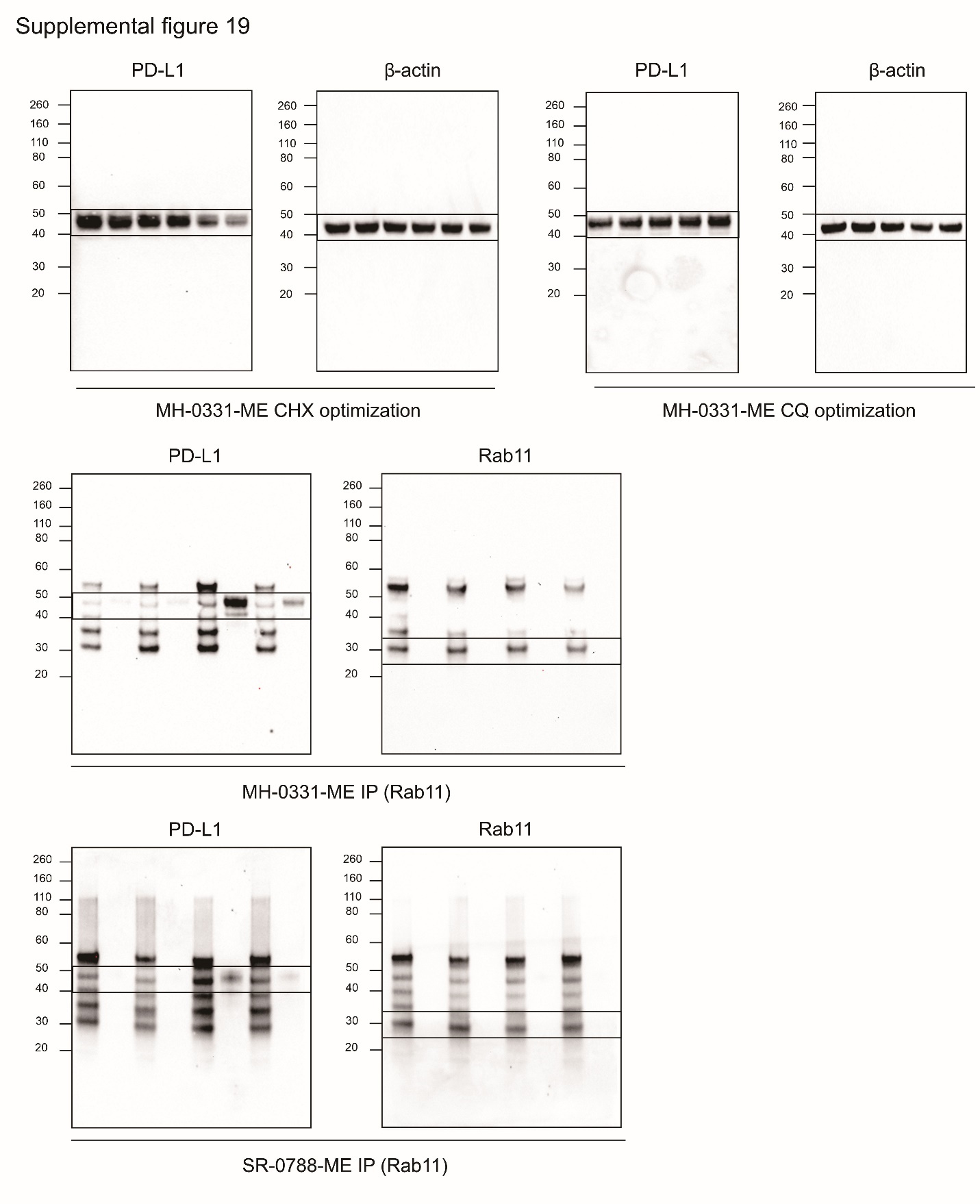


**Uncropped western blot images.** Uncropped western blot images for Figure S3A-C.


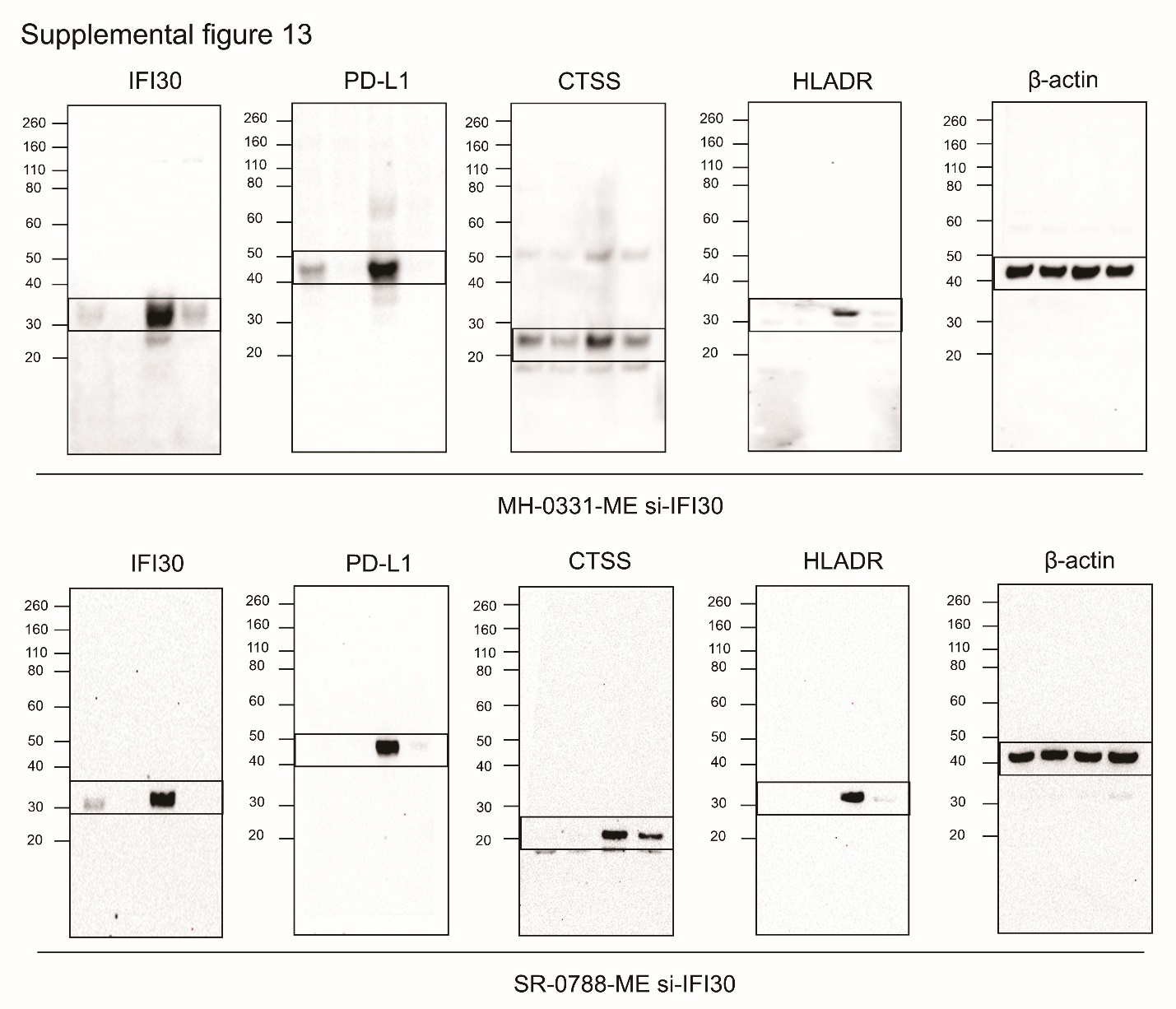


**Uncropped western blot images.** Uncropped western blot images for Figure S5B.


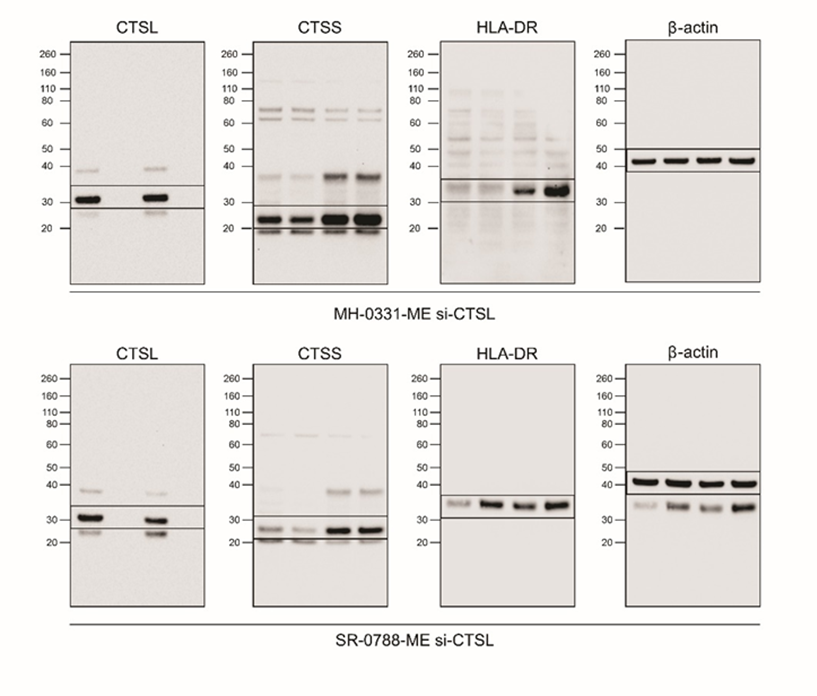


**Uncropped western blot images.** Uncropped western blot images for Figure S5C.


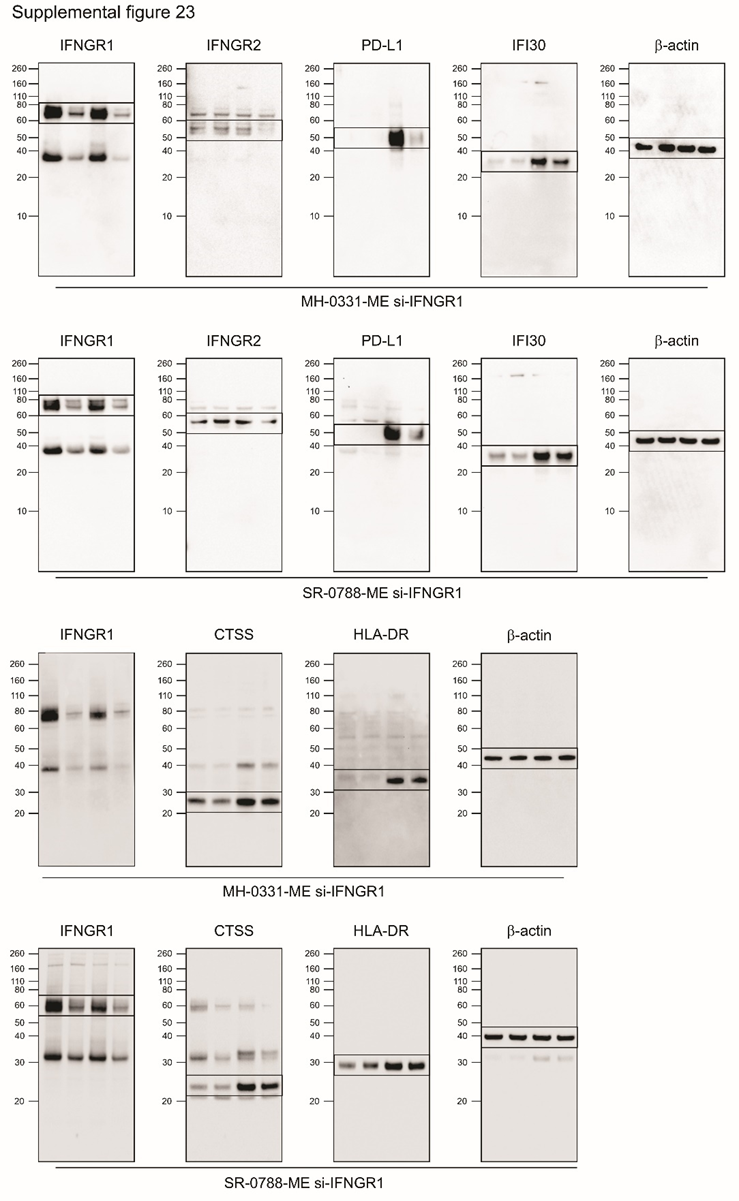


**Uncropped western blot images.** Uncropped western blot images for Figure S5D.
